# Preliminary establishment of genetic transformation system for embryogenic callus of *Acer truncatum* ‘Lihong’

**DOI:** 10.3389/fpls.2024.1419313

**Published:** 2024-09-05

**Authors:** Yipeng Yang, Yuan Chan, Yongge Wang, Hao Guo, Lina Song, Huali Zhang, Liping Sun, Richen Cong, Hua Zhang

**Affiliations:** Beijing Key Laboratory of Greening Plants Breeding, Beijing Academy of Forestry and Landscape Architecture, Beijing, China

**Keywords:** *Acer truncatum* ‘Lihong’, GST894 gene, regeneration system, embryonic callus, genetic transformation system

## Abstract

**Introduction:**

*Acer truncatum* Bunge, belonging to the Acer genus in the Aceraceae family, is a commonly planted afforestation species across China, Japan, Korea, Europe, and North America. Renowned for its vibrant fall colors, it holds significant ecological and ornamental value.

**Methods:**

In this study, *Acer truncatum* ' Lihong ' was used as the research object. Starting from the callus induction of explants, the embryogenic callus of *Acer truncatum* 'Lihong' was obtained by systematically optimizing the medium and culture conditions. Then, the candidate gene AtrGST894 screened by transcriptome sequencing was transformed into embryogenic callus by Agrobacterium-mediated transformation. The genetic transformation system of *Acer truncatum* 'Lihong' embryogenic callus was initially established by continuously adjusting the conditions of Agrobacterium tumefaciens infection receptor materials, thus laying a material foundation for the study of the molecular regulation mechanism of *Acer truncatum* 'Lihong' leaf color, and also preparing for the later molecular improvement breeding of *Acer truncatum*. Therefore, this study has important theoretical and practical significance.

**Results:**

The results showed that the best medium for callus induction of *Acer truncatum* was 1/2MS+2 mg/L 2,4-D+0.3 mg/L 6-BA+0.5 mg/L NAA; The embryogenic callus induction medium of *Acer truncatum* was 1/2MS+3.0mg/L 6-BA+2.0mg/L TDZ+0.5mg/L IBA+0.1mg/L GA_3_; The proliferation medium of embryogenic callus of *Acer truncatum* was WPM+1.0mg/L TDZ+0.5mg/L IBA+0.1mg/L GA_3_+3mg/L 6-BA+1.0mg/L KT; The infection experiment of Agrobacterium tumefaciens on the embryogenic callus of *Acer truncatum* showed that the best antibacterial medium was WPM+30g/L sucrose+8g/L agar+0.5g/L acid-hydrolyzed casein+0.2mg/L KT+1.0 mg/L TDZ+0.5 mg/L IBA+0.1 mg/L GA_3_+200mmol/L carboxybenzyl+200mg/L cephalosporin, and then WPM+30g/L sucrose+8g/L agar+0.5g/L acid-hydrolyzed casein+0.2mg/L KT+1.0 mg/L TDZ+0.5 mg/L IBA+0.1 mg/L GA_3_+300mmol/L carboxybenzyl+200mg/L cephalosporin+25mg/L hygromycin. Screening medium screening, The obtained embryogenic callus browning rate, pollution rate and mortality rate were the lowest, and maintained vigorous growth.

**Discussion:**

The embryogenic callus was used as the infection material to verify that we successfully transferred the target gene into the embryogenic callus, which means that the genetic transformation system of *Acer truncatum* embryogenic callus was partially completed, and the infection process could be effectively inhibited. Although there was partial browning, it could continue to proliferate. Therefore, in future experiments, the focus is still to continue to verify the optimal conditions for optimizing the genetic transformation of *Acer truncatum* embryogenic callus and to solve the problems of difficulty in embryonic callus germination.

## Introduction

*Acer truncatum* Bunge, belonging to the *Acer* genus in the *Aceraceae* family, is a commonly planted afforestation species across China, Japan, Korea, Europe, and North America. Renowned for its vibrant fall colors, it holds significant ecological and ornamental value (Ma et al., 2020). After over 20 years of variety breeding, our *Acer truncatum* Bunge breeding team has successfully developed a new variety named *Acer truncatum* ‘Lihong’. This cultivar consistently displays striking red foliage in the fall, particularly suited for low-altitude urban areas of Beijing. *Acer truncatum* ‘Lihong’ leaves transform into a blood-red hue from late October to mid-November, enhancing its ornamental value.

Changes in plant leaf color occur due to alterations in pigment content within the plant. These pigments primarily include chlorophyll, carotenoids, and anthocyanidins (Li et al., 2023). Notably, red color predominantly relies on anthocyanidins, vital plant pigments contributing to a diverse range of colors, from orange and red to violet and blue (Tanaka and Ohmiya, 2008). Among the 20 anthocyanidin classes identified, 6 are commonly found in plants: pelargonidin, cyanidin, delphinidin, peonidin, petunidin, and malvidin. Anthocyanidin synthesis and accumulation are intricate processes regulated by various enzyme genes and transcription factors. Additionally, external environmental factors such as temperature, light, and different stresses also influence this process (Wang J. et al., 2022).

Numerous studies have elucidated the biosynthetic pathway of anthocyanidins, a subset of the phenylpropanoid biosynthetic pathway widely conserved across plant species. Key structural genes involved in both early and late anthocyanidin synthesis include phenylalanine ammonia-lyase (*PAL*), chalcone synthase (*CHS*), chalcone isomerase (*CHI)*, flavanone 3-hydroxylase (*F3H*), flavonoid 3’-hydroxylase (*F3’H*), flavonoid 3’,5’-hydroxylase (*F3’5’H*), dihydroflavonol 4-reductase (*DFR*), anthocyanidin synthase (*ANS*), and UDP-glucose flavonoid 3-O-glycosyltransferase (*UFGT*) (Le Maitre et al., 2019). These genes have been extensively studied in various plant species, including Arabidopsis thaliana (Pietrowska-Borek and Katarzyna, 2013), maize (Dooner, 1983), apple (Wang X. et al., 2022), poplar (Wu et al., 2020), grapevine (Guo et al., 2022), and potato (Odgerel et al., 2022). Researchers have analyzed their mechanisms of action. Modulating the expression levels of these structural genes can induce changes in anthocyanidin species and content, resulting in varying colors among different plant species, genotypes, organs, and even different locations of the same plant. For instance, UDP-glucose:flavonoid 3-O-glycosyltransferase (UFGT) is a key enzyme for anthocyanidin synthesis in American Acer rubrum (Feng et al., 2008).

Anthocyanidins are synthesized in the cytoplasm and then transported into vesicles for accumulation and visualization through transporter proteins across the vesicular membrane (Liu et al., 2019). Transporter proteins associated with anthocyanidin transport, such as glutathione S-transferases (GSTs), have been identified in various plant species, including kiwifruit (Liu et al., 2019), peach (Zhao et al., 2020), and cotton (Chai et al., 2022), indicating their conserved role in anthocyanidin accumulation. Studies have revealed that most GSTs are involved in transporting either cyanidin or delphinidin. Currently, GST proteins involved in anthocyanidin transport have been isolated and identified in plants such as *Arabidopsis thaliana*, apple, and grape (Kitamura et al., 2010; Jiang et al., 2019; Poudel et al., 2021).

Through transcriptome and metabolome analyses conducted at various stages of color change, researchers have investigated candidate genes and metabolic pathways underlying leaf color change in *Acer mandshuricum* Maxim., elucidating its color mechanism. This research serves as a theoretical basis for genetic improvement and breeding of *Acer mandshuricum*. In a separate study, researchers performed *de novo* transcriptome assembly, annotation, and bioinformatics analysis on leaves of *Acer pseudosieboldianum* (Pax) Kom. during different color change phases in autumn. They identified and validated differential genes and specified those associated with anthocyanidin synthesis, corroborating these findings with anthocyanidin metabolism data. This investigation revealed five anthocyanidins involved in leaf color change in *Acer pseudosieboldianum*, with cyanidin notably influencing the final leaf color (Gao et al., 2021).

Most studies on plant anthocyanidin metabolism have focused on horticultural plants such as apples, grapes, strawberries, chrysanthemums, lilies, moonflowers, and hyacinths. However, investigations concerning Aceraceae Juss. have predominantly focused on metabolome analysis and transcriptome gene screening, with limited detailed analyses of gene functions. Consequently, there remains a lack of understanding regarding anthocyanidin species, crucial structural/modification/transporter genes, transcriptional complexes, and their upstream regulators in this plant family.

To address this gap, candidate genes related to plant traits can be explored through multi-omics investigations and association analyses, followed by functional studies. The most reliable identification method involves genetic transformation within plant ontology to perform functional analysis of candidate genes. Efficient genetic transformation systems have been established in various plants such as *Populus*L (Zhu et al., 2015), *Ziziphus jujube* (Cui et al., 2018), *Solanum tuberosum* L (Zhang et al., 2019), *Setaria italica* (Li et al., 2019), *Camellia sinensis* (L.) (Wang, 2021), and *Catalpa bungei* (Cen et al., 2021), facilitating subsequent gene function studies and improved breeding.

The enhancement of the rice genetic transformation system is crucial for breeding disease-resistant, herbicide-resistant, drought-resistant, high-yielding, high-quality, and nutrient-efficient rice varieties (Li et al., 2012). However, efficient genetic transformation systems are lacking for most woody plants, particularly trees. This study focuses on *Acer truncatum* ‘Lihong’ to develop a genetic transformation system for *Acer truncatum* ‘Lihong’ using exosome-induced callus. This involves systematically optimizing the medium and cultivation conditions and adjusting *Agrobacterium*-infected receptor materials. The developed system will facilitate the functional verification of candidate genes associated with leaf color, establish a material foundation for investigating the molecular regulatory mechanism of *Acer truncatum* ‘Lihong’ leaf color, and prepare for future molecular improvement of *Acer truncatum* Bunge breeding. Hence, this study holds significant importance.

## Materials and methods

### Transcriptomic analysis of *Acer truncatum* leaves

The leaf samples of common *Acer truncatum* Bunge (CK) and *Acer truncatum* ‘Lihong’ (LH) from five different periods were rapidly frozen in liquid nitrogen and then placed on dry ice for shipment to Biomarker Technologies Corporation in Beijing for total RNA extraction. The sampling process for both common *Acer truncatum* Bunge and *Acer truncatum* ‘Lihong’ is shown in **Supplementary Figure 1.**

To obtain high-quality data, the raw data needs to be filtered to generate clean data. The Trinity software is used for sequence assembly, resulting in the Transcript and Unigene libraries of Acer truncatum. The Unigene sequences are then aligned with gene sequences from various databases to obtain annotation information. To identify differentially expressed genes (DEGs) between common *Acer truncatum* Bunge (CK) and *Acer truncatum* ‘Lihong’ (LH) during the five color change periods, a false discovery rate (FDR) < 0.01 and fold change > 2 are used as screening criteria. Firstly, based on the preliminary screened DEGs between common *Acer truncatum* Bunge (CK) and *Acer truncatum* ‘Lihong’ (LH), genes related to anthocyanin synthesis, modification, and transport are further selected based on annotation information. Genes with an average expression level greater than 20 across the periods are used as a selection criterion. The expression data provided by the transcriptome is then used to analyze the differences between the five comparison groups (CK1 vs. LH1, CK2 vs. LH2, CK3 vs. LH3, CK4 vs. LH4, CK5 vs. LH5), screening for candidate genes that show significantly higher expression in *Acer truncatum* ‘Lihong’ than in *Acer truncatum* Bunge during the same period.

By analyzing the expression levels and significance of differences in anthocyanin-related differentially expressed genes (DEGs) between common Acer truncatum (CK) and Acer truncatum ‘Lihong’ (LH) across five periods in the transcriptomic data, the key gene *GST894* (LG05.1609) was identified. The reliability of the data was verified using qRT-PCR.

### Primary callus induction

Seeds of *Acer truncatum* ‘Lihong’ exhibiting vigorous growth in the nursery site of the Beijing Institute of Landscape Architecture were collected annually from 2020 to 2022 and sterilized. The treated seeds were then inoculated on ½ Murashige and Skoog medium (½ MS basal medium). Subsequently, they were cultured in the dark at 15°C for 1 week and germinated for 3-5 weeks until they grew into 5-7 cm seedlings (**Supplementary Figure 2**).

The basal medium utilized in this experiment comprised ½ MS and woody plant medium (WPM) (**Supplementary Tables 1**, **2**). The temperature of the culture room was 24 ± 2°C, with a cycle was 16-h/8-h(light/dark) and a light intensity of approximately 5000 Lux. *Agrobacterium* GV3101 competence and the plasmid pCAMBIA1305, a vector offering hygromycin (Hygromycin B) resistance, were used.

Sterilized outdoor young stem segments, leaves, and sterile seedling stem segments of *Acer truncatum* ‘Lihong’ were selected as inoculation materials. After inoculation, they were transferred to the culture room for light cultivation. In this phase of the experiment, ½ MS served as the basal medium, along with two hormone combinations: formulation 1: 0.01 μmol L^-1^ thidiazuron (TDZ) + 2.0 mg L^-1^ 2,4-dichlorophenoxyacetic acid (2,4-D) formulation 2: 2.0 mg L^-1^ 2,4-D + 0.3 mg L^-1^ 6-benzylaminopurine (6-BA) + 0.5 mg L^-1^ 1-naphthaleneacetic acid (NAA).

### Embryonic callus induction and identification

The primary callus was inoculated with various hormone combinations on ½ MS as the basal medium to induce embryonic callus production, with specific hormone concentrations and ratios detailed in **Supplementary Table 3**. A small portion of the resulting callus was fixed using FAA fixative and embedded in resin to prepare ultrathin sections after vacuum pumping. These ultrathin sections were then examined and photographed under a microscope to identify the type of embryonic callus.

### Embryonic callus proliferation

Embryonic callus proliferation was induced using WPM as the basal medium. 2 specific hormone formulations were employed:

**Effect of 11-ketotesterone (KT) concentration on embryogenic callus proliferation:** The medium was supplemented with 1.0 mg L^-1^ TDZ, 0.5 mg L^-1^ indole-3-butyric acid (IBA), and 0.1 mg L^-1^ gibberellic acid (GA_3_). Different concentrations of KT (0.1, 0.2, 0.5, 1.0, and 2.0 mg L^-1^) were then added, and embryogenic callus proliferation was observed.

**Effect of the combination of 6-BA and KT on embryogenic callus proliferation:** The medium was supplemented with 1.0 mg L^-1^ TDZ, 0.5 mg L^-1^ IBA, and 0.1 mg L^-1^ GA_3_. Healthy embryogenic callus was inoculated on combinations of 6-BA and KT: 2 mg L^-1^ 6-BA + 0.5 mg L^-1^ KT; 2 mg L^-1^ 6-BA + 1.0 mg L^-1^ KT; 3 mg L^-1^ 6-BA + 0.5 mg L^-1^ KT; 3 mg L^-1^ 6-BA + 1.0 mg L^-1^ KT. Embryogenic callus proliferation was then observed.

### Cloning of the *GST894* gene and vector construction

In this experiment, the pCAMBIA1305.1-GFP vector was selected for constitutive expression. The plasmid map is shown in **Supplementary Figure 3**, where all marked points indicate single cloning sites.

According to the manufacturer’s instructions, total RNA was isolated from the leaves of ‘Lihong’ Acer truncatum using the RNAprep Pure Plant Kit (TIANGEN). Gene-specific primers (F: ATGGCAGGCATCAAAAT; R: CTTCTTGCTTTGCAAAG) were used for the polymerase chain reaction (PCR) with DNA polymerase (Vazyme) in a total volume of 50 μL. The initial denaturation step was performed at 94°C for 3 minutes, followed by 34 cycles of 94°C for 45 seconds, 58°C for 30 seconds, and 72°C for 90 seconds, with a final extension step at 72°C for 5 minutes. The amplified *GST894* product was then inserted into the pCAMBIA1305.1-GFP vector.

### Embryonic callus transformation

In this experiment, the pCAMBIA1305.1-GFP vector was selected for transformation, with the plasmid map shown in **Supplementary Figure 3**. According to the instructions, the vector was transformed into Agrobacterium tumefaciens strain GV3101.

**Pre-culture:** Vigorously growing embryonic callus was cut into small pieces (0.5 cm^3^, Sample A; 0.5 cm^3^, Sample B; 1 cm^3^, Sample C) and placed on pre-culture medium in darkness (2 d for A, 2 d for B, and 1 d for C). The medium formulations were as follows: (**Supplementary Table 4**)

**Infestation:** *Agrobacterium* transformed with the target plasmid was proliferated to OD_600_ = 0.6–0.8 and then centrifuged to collect the thallus. After discarding the supernatant, the thallus was resuspended in liquid medium [½ MS (A), WPM (B), and WPM (C)] and collected by centrifugation at 4000 rpm for 10 min. The supernatant was discarded, and the process was repeated thrice. Subsequently, 100 μmol L^-1^ acetosyringone (AS) and 0.025% Silwet-77 were added. After shaking, a sterile wide-mouth jar containing *Acer truncatum* ‘Lihong’ embryonic callus and resuspended bacterial solution was gently shaken at 100 rpm for 10 min at 28°C on a shaker.

**Co-culture:** Inoculation was performed in a co-culture medium followed by dark culture at 25°C [60 h (A), 48 h (B), 48 h (C)]. The specific medium formulations were as follows: (**Supplementary Table 5**).

**Sterilization:** The samples were first washed with sterile water for 2 min, then thrice with specific liquid media, 200 mg L^-1^ carbenicillin, and 400 mg L ^-1^cephalosporin, and finally with sterile water.

**Antibacterial culture:** The embryogenic callus was inoculated with 200 mg L^-1^ carbenicillin and 200 mg L^-1^ cephalosporin in the medium for light culture. The specific medium formulations were as follows: (**Supplementary Table 6**).

**Resistance screening:** The medium (WPM + 30 g L^-1^ sucrose + 8 g L^-1^ agar + 0.5 g L^-1^ acid hydrolyzed casein + 3 mg L^-1^ 6-BA + 1.0 mg L^-1^ TDZ + 0.5 mg L^-1^ IBA + 0.1 mg L^-1^ GA_3_) was supplemented with 300 mg L^-1^ carbenicillin, 200 mg L^-1^ cephalosporin, and hygromycin for positive embryogenic callus screening. 4 concentration gradients of hygromycin were used, and the survival rate was assessed after 7 days of incubation (**Supplementary Table 7**).

### Transgene positive embryogenic callus identification

Simultaneously extract total RNA from both infected and uninfected embryogenic callus tissues. Follow the instructions of the Vazyme cDNA Reverse Transcription Kit. Use the Acer truncatum actin gene as an internal control, designing upstream and downstream primers. Adjust the amount of cDNA added based on the brightness of the actin band on the gel until the amplified actin brightness is consistent. Design upstream and downstream primers for the target gene based on its sequence (**Supplementary Table 8**). Use the cDNA from infected and uninfected embryogenic callus tissues as templates and amplify the gene sequence using Fast Taq DNA Polymerase.

Use fluorescence observation to further identify whether the embryogenic callus has integrated the target gene. Detect GFP fluorescence in the embryogenic callus tissues using a stereomicroscope. The embryogenic callus tissues where green fluorescent spots can be observed are transgenic embryogenic callus tissues.

### Expression of *GST894* gene in transgenic embryonic callus tissues

Take uninfected embryonic callus tissue, embryonic callus tissue transformed with the empty vector, and embryonic callus tissue transformed with the *GST894* gene. Grind them separately in liquid nitrogen, extract RNA, and reverse transcribe to obtain cDNA. Use real-time qPCR to detect changes in the expression levels of the *GST894* gene in transgenic embryonic callus tissues, using the Acer truncatum actin gene as the internal reference gene. Follow the reaction system and procedure as per the instructions of the Vazyme qRT-PCR kit. Calculate the relative expression levels using the 2^-△△CT^ method. Plot the results using Origin 2019b, with three biological replicates.

### Statistical analysis


$$\text{Callus induction rate=}\frac{\text{Number of explants inducing callus}}{\text{Total number of inoculated explants}}\times 100\%$$

$$\text{Induction survival rate=}\frac{\text{Number of surviving calli induced}}{\text{Total number of inoculated calli}}\times 100\%$$

$$\text{Browning rate=}\frac{\text{Number of browned calli}}{\text{Total number of inoculated calli}}\times 100\%$$

$$\text{Antibacterial rate=}\frac{\text{Number of aseptic calli}}{\text{Total number of inoculated calli}}\times 100\%$$

$$\text{Survival rate=}\frac{\text{Number of surviving calli}}{\text{Total number of inoculated calli}}\times 100\%$$

$$\text{Proliferation coefficient=}\frac{\text{Number of calli segments after proliferation}}{\text{Number of inoculated calli before proliferation}}\times 100\%$$

Note: Volume of calli segments after proliferation = Volume of calli before inoculation

The data was processed using Microsoft Office Excel 2019 and IBM SPSS Statistics 26.0 software. One-way analysis of variance (ANOVA) was performed using the Duncan test.

## Results

### Results of sequencing data output

In this experiment, 10 cDNA libraries were constructed using leaf samples from two Acer truncatum varieties at five different color-changing periods. The sequencing data output is shown in **Supplementary Table 9**. The number of clean reads ranged from 20,137,758 to 23,835,267. The GC content was approximately 45%. The percentage of Q20 bases was over 97%, and the percentage of Q30 bases was over 92%, indicating that the sequencing data met the standards and was of high quality, suitable for subsequent experiments (**Supplementary Table 9**).

### Sequencing data assembly results

After assembling the clean reads, 303,592 transcripts and 110,872 unigenes were obtained. The length distribution of the unigenes and transcripts is shown in **Supplementary Table 10**. The majority, approximately 31.11%, are longer than 2000 nt, while the smallest proportion, 12.65%, falls within the 300-500 nt range. The average length is 1628.62 nt, with an N50 of 2680 nt. As shown in **Supplementary Table 10**, the number of unigenes decreases as their length increases, with an average length of 754.71 nt and an N50 of 1389 nt.

### Functional annotation and classification of unigenes

The 110,872 unigene sequences assembled from Acer truncatum were compared with sequences in various functional databases (COG, GO, KEGG, KOG, Pfam, Swissprot, eggNOG, NR) to obtain functional annotations. The annotation results, as shown in **Supplementary Table 11**, indicated that 73,673 unigene sequences were successfully annotated, accounting for approximately 66.45% of the total unigenes.

A total of 52,848 unigenes were annotated in the GO database, with the annotation results shown in **Supplementary Figure 4**. The “Cellular Component” category was divided into 15 functional groups. The group with the most annotations was “cell” (27,065 unigenes), followed by “cell part” (26,993 unigenes), and “membrane” (21,734 unigenes).In the “Molecular Function” category, which was divided into 15 functional groups, the most annotated unigenes were found in “catalytic activity” (28,673 unigenes) and “binding” (24,880 unigenes).The “Biological Process” category included 20 functional groups. The top three groups with the most unigene annotations were “metabolic process” (28,906 unigenes), “cellular process” (25,532 unigenes), and “single-organism process” (20,339 unigenes). Overall, the three major categories were divided into 50 functional groups, with individual unigenes potentially being annotated in multiple groups.

In the GO database annotation information, among the 17 Unigenes related to anthocyanins or proanthocyanidins, 12 are annotated under the ‘biological process’ category, Among them, the ‘anthocyanin compound biosynthetic process’ functional group mainly includes 5 Unigenes; The ‘regulation of anthocyanin biosynthesis’ functional group includes 1 Unigene; Each of the functional groups ‘anthocyanin compound metabolism’, ‘proanthocyanidin biosynthesis’, and ‘accumulation of anthocyanins in tissues under UV light’ includes 2 Unigenes; Additionally, there is 1 Unigene annotated as ‘negative regulation of anthocyanin metabolism’. In the ‘molecular function’ category, the functional group ‘glucosyltransferase activity’ includes 3 Unigenes; In the ‘anthocyanin reductase activity’ functional group, 2 Unigenes are annotated.

### Differential gene expression analysis

By analyzing the leaves of common *Acer truncatum* Bunge (CK) and ‘ *Acer truncatum* ‘Lihong (LH), a total of 11,984 differentially expressed genes (DEGs) were identified, of which 7,585 DEGs were related to the flavonoid biosynthesis pathway associated with autumn leaf coloration. Comparison between CK1 and LH1 revealed 985 DEGs, with 508 upregulated and 477 downregulated. Between CK2 and LH2, there were 1,720 DEGs, with 883 upregulated and 837 downregulated. Between CK3 and LH3, there were 1,251 DEGs, with 755 upregulated and 496 downregulated. Between CK4 and LH4, there were 1,618 DEGs, with 795 upregulated and 823 downregulated. Comparison between CK5 and LH5 showed 2,900 DEGs, with 1,647 upregulated and 1,253 downregulated. By drawing a Venn diagram (**Supplementary Figure 5**), it was found that there were 125 common DEGs across the five groups.

### Identification and screening of genes related to the anthocyanin biosynthesis pathway

The synthesis of anthocyanins is an extremely complex process that may involve various structural genes and transcriptional regulators from generation to modification, and finally, the transport to vacuoles. By organizing the data provided by the transcriptome, genes involved in the anthocyanin metabolic pathway were screened, as shown in **Supplementary Figure 6**. A total of 59 genes related to anthocyanin synthesis were identified. Among them, GST genes were the most numerous, with 16 genes. These genes generally showed higher expression levels in CK1, CK2, LH1, and LH2, with significantly higher expression in LH compared to CK.

### Gene qRT-PCR validation

The qRT-PCR experimental results for gene *GST894* showed that the expression level of *GST894* was higher in LH at all stages compared to CK (**Supplementary Figure 7**). Therefore, functional validation of gene *GST894* was performed.

### Primary callus induction

**Figure 1:**
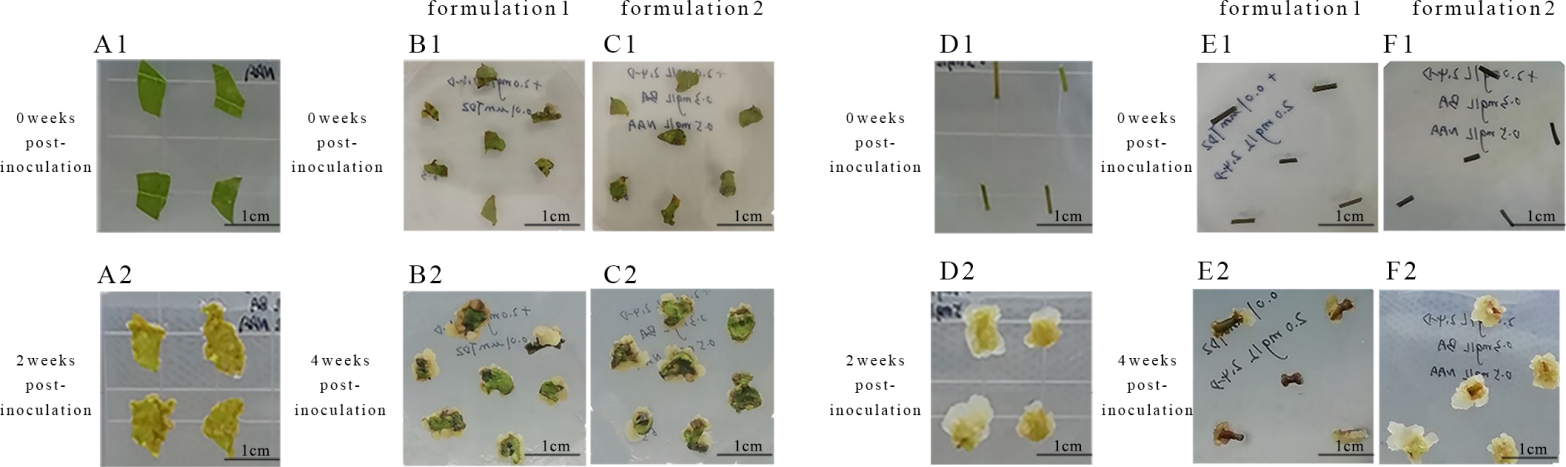
Primary callus induction in *Acer truncatum* ‘Lihong’ **(A1)** Aseptic seedling leaf inoculation; **(A2)** Aseptic seedling leaf inoculation for 2 weeks ; **(B1)** Formula 1 leaf inoculation; **(B2)** Formula 1 leaf inoculation for 4 weeks; **(C1)** Formula 2 leaf inoculation; **(C2)** Formula 2 leaf inoculation for 4 weeks; **(D1) **Aseptic seedling stem segment inoculation; **(D2) **Aseptic seedling stem segment inoculation for 2 weeks; **(E1) **Formula 1 stem segment inoculation; **(E2)** Formula 1 stem segment inoculation for 4 weeks; **(F1) **Formula 2 stem segment inoculation; **(F2) **Formula 2 stem segment inoculation for 4 week.

After 3 d of inoculation, stem segments exhibited expansion at both ends, with callus appearing at the incisions of individual stem segments. After 1 week, the callus formation rate of stem segments reached 88.3% (**Table 1**), while leaves exhibited minimal callus induction, with most showing signs of white or browning and necrosis (**Figure 1**). Consequently, stem segments from tissue culture seedlings proved to be the most suitable explants for inducing *Acer truncatum* ‘Lihong’ callus.

**Table 1:** Callus induction of different sources of stem segments and leaves.

Material Sources	Tissue Site	Callus Induction Rate (%)		Callus Condition	Biological Replicates
		1 week	2 weeks		
Outdoor Material	Stem segment	77.8 ± 0.06b	88.9 ± 0.35b	Mostly yellow-green, white, or yellow; some are compact and dense granules, few are agglomerated.	3
	Leaves	0.0 ± 0.00c	44.5 ± 0.19d	Essentially a yellow mass.	3
Sterile Seedling	Stem segment	88.3 ± 0.17a	100.0 ± 0.00a	Mostly yellow-green, tender red, or yellow; tight, dense, crystalline granules.	3
	Leaves	0 ± 0.00c	55.3 ± 0.12c	Some white and crystalline callus; and a few yellow masses, less compact and amorphous.	3

Values in the same column followed by different lowercase letters indicate significant differences (P < 0.05).

Observations of callus growth induced by leaf and stem explants on different formulations revealed that 2,4-D induced compact, dense, and predominantly light-green callus. Notably, the callus formation rate of formulation 2 stem segments significantly exceeded that of formulation 1 (**Table 2**). These results indicate that formulation 2 was the most effective, yielding callus with a preferable green granular appearance conducive to differentiation (**Figure 1**).

**Table 2:** Effect of different culture media on callus induction.

Explant	Culture Medium	Induction Rate (%)	Biological Replicates
Sterile Stem	Formulation 1	77.6 ± 0.28c	3
	Formulation 2	100 ± 0.00a	3
Sterile Leaves	Formulation 1	76.4 ± 0.39c	3
	Formulation 2	89.2 ± 0.26b	3

Values in the same column followed by different lowercase letters indicate significant differences (P < 0.05).

### Embryonic callus induction and identification

**Figure 2:**
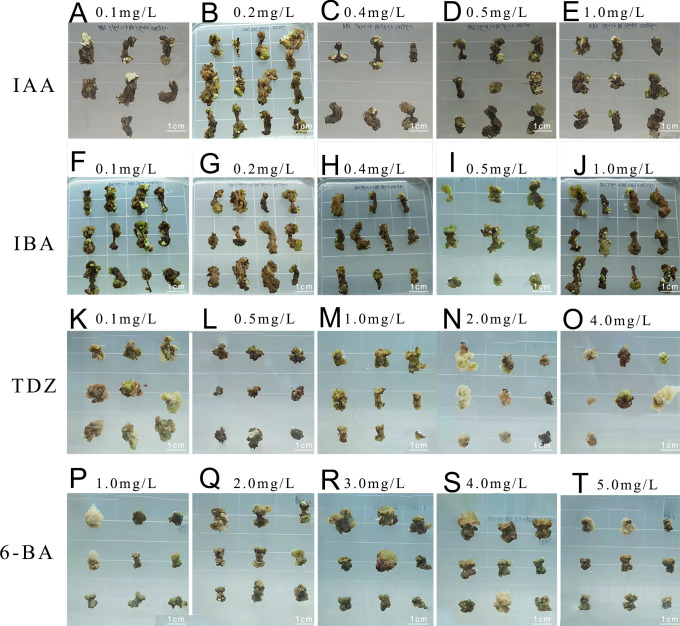
Embryonic callus induction **(A-E) **Callus growth conditions on medium with 0.1, 0.2, 0.4, 0.5, and 1.0 mg L^-1^ IAA concentration, respectively; **(F-J)** Callus growth conditions on medium with 0.1, 0.2, 0.4, 0.5, and 1.0 mg L^-1^ IBA concentration, respectively; **(K-O)** Callus growth conditions on medium with 0.1, 0.5, 1.0, 2.0, and 4.0 mg L^-1^ TDZ concentration, respectively; **(P-T)** Callus growth conditions on medium with 1.0, 2.0, 3.0, 4.0, and 5.0 mg L^-1^ 6-BA concentration, respectively.

The addition of IAA to the medium resulted in slow callus growth and continued browning during subsequent culture (**Table 3**), indicating inferior performance compared to the medium supplemented with IBA (**Figures 2A–E**). One-way ANOVA revealed that the induction survival rate in Medium No. 9 was markedly higher than in other treatment groups (*P* < 0.05), with favorable callus texture. This suggests that adding 0.5 mg L^-1^ of IBA substantially improved the induction survival rate of embryonic callus under similar conditions (**Figures 2F–J**).

**Table 3:** Effect of different concentrations of IAA and IBA on embryonic callus formation.

Medium No.	Plant Hormone (mg L^-1^)					Callus Condition			Biological Replicates
	IAA	IBA	TDZ	6-BA	GA_3_	Induction Survival Rate (%)	Browning/Mortality Rate (%)	Callus Texture	
1	0.1	0.0	1.0	3.0	0.1	42.5 ± 4.092a	57.50 ± 4.09c	Yellowish-brown and denser	3
2	0.2	0.0	1.0	3.0	0.1	32.65 ± 2.376ab	67.35 ± 2.38bc	Yellowish-brown and denser	3
3	0.4	0.0	1.0	3.0	0.1	25.83 ± 5.973cb	74.17 ± 5.97ab	Yellowish-brown and less compact	3
4	0.5	0.0	1.0	3.0	0.1	19.17 ± 2.541c	80.84 ± 2.54a	Yellowish-brown and less compact	3
5	1.0	0.0	1.0	3.0	0.1	30.55 ± 4.17acb	69.45 ± 4.17abc	Yellowish-brown and dense	3
6	0.0	0.1	1.0	3.0	0.1	60.48 ± 7.93b	39.52 ± 7.93a	Yellowish-brown and denser	3
7	0.0	0.2	1.0	3.0	0.1	50.77 ± 2.61b	49.23 ± 2.61a	Yellow-green and denser	3
8	0.0	0.4	1.0	3.0	0.1	62.54 ± 5.50b	37.46 ± 5.50a	Yellow-green and less compact	3
9	0.0	0.5	1.0	3.0	0.1	82.31 ± 5.26a	17.70 ± 5.26b	Yellow-green and less compact	3
10	0.0	1.0	1.0	3.0	0.1	63.89 ± 6.26b	36.11 ± 6.26a	Yellow-green and denser	3

Values in the same column followed by different lowercase letters indicate significant differences (P < 0.05).

Adjusting the TDZ concentration led to improved embryonic callus induction rates across all treatments (**Figures 2K–O**). According to the statistical analysis (**Table 4**), when the TDZ concentration reached 2 mg L^-1^, the callus exhibited a granular and compact texture, resulting in a significantly higher induction survival rate than other treatment groups with similar callus status (*P* < 0.05).

**Table 4:** Effect of different concentrations of TDZ on embryonic callus formation.

Medium No.	Plant Hormone (mg L^-1^)				Callus Condition			Biological Replicates
	TDZ	IBA	6-BA	GA_3_	Induced Survival rate (%)	Browning/Mortality Rate (%)	Callus Texture	
1	0.1	0.5	3.0	0.1	74.56 ± 4.9b	25.44 ± 4.90a	Yellow-green, granular, and less compact	3
2	0.5	0.5	3.0	0.1	66.06 ± 1.74b	33.94 ± 1.74a	Yellowish-brown, agglomerated, and dense texture	3
3	1.0	0.5	3.0	0.1	75.63 ± 2.67b	24.38 ± 2.67a	Yellowish white, granular, and dense texture	3
4	2.0	0.5	3.0	0.1	90.79 ± 3.23a	9.22 ± 3.23b	Yellow-green, granular, and dense texture	3
5	4.0	0.5	3.0	0.1	76.6 ± 5.72b	23.4 ± 5.72a	Yellow-green, agglomerated, and dense texture	3

Values in the same column followed by different lowercase letters indicate significant differences (P < 0.05).

Furthermore, the induction rate increased with increasing 6-BA concentration, peaking at 3 mg L^-1^ (**Figures 2P–T**). The survival rate of embryonic callus induction in the medium containing 3 mg L^-1^ 6-BA was markedly higher than in other treatment groups (**Table 5**).

**Table 5:** Effect of different concentrations of 6-BA on embryonic callus formation.

Medium No.	Plant Hormone (mg L^-1^)				Callus Condition			Biological Replicates
	TDZ	IBA	6-BA	GA_3_	Induced Survival rate (%)	Browning/Mortality Rate (%)	Callus Texture	
1	2.0	0.5	1.0	0.1	85.14 ± 2.67b	14.86 ± 2.67b	White-green, granular, and less compact	3
2	2.0	0.5	2.0	0.1	87.65 ± 1.00b	12.35 ± 1.00b	Yellow-green, granular, and dense texture	3
3	2.0	0.5	3.0	0.1	96.05 ± 0.50a	3.95 ± 0.50c	Yellow-green, granular, and dense texture	3
4	2.0	0.5	4.0	0.1	87.56 ± 1.89b	12.44 ± 1.89b	Yellow-green, granular, and dense texture	3
5	2.0	0.5	5.0	0.1	76.35 ± 1.66c	23.65 ± 1.66a	Yellowish-white, granular, and dense texture	3

Values in the same column followed by different lowercase letters indicate significant differences (P < 0.05).

### Identification of embryonic callus in *Acer truncatum* ‘Lihong’

**Figure 3:**
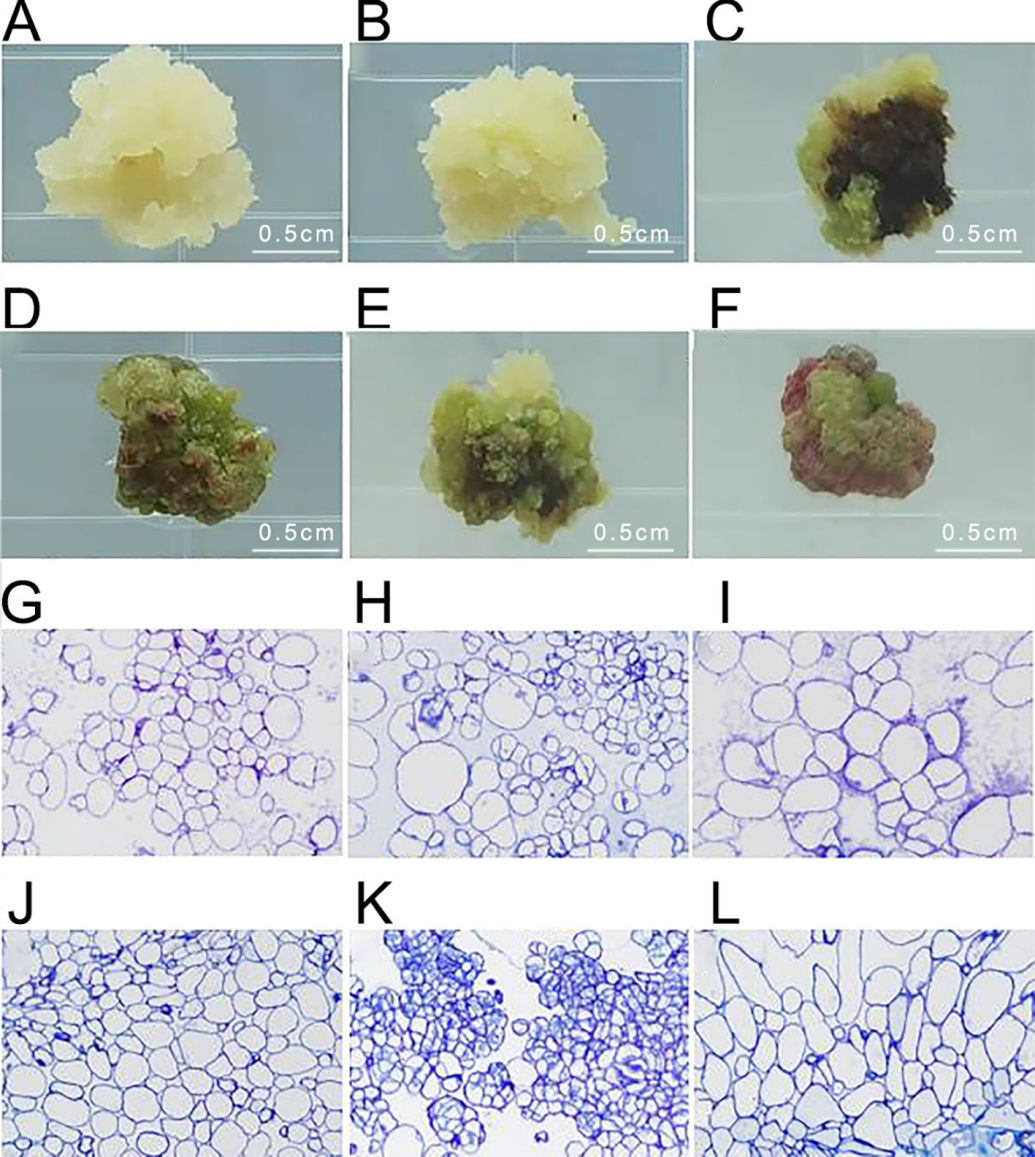
Identification of embryonic callus in *Acer truncatum* ‘Lihong’ **(A)** White fluffy callus; **(B)** White granular callus; **(C)** Green loose callus; **(D)** Green compact callus; **(E)** Green granular callus; **(F)** Red granular callus; **(G-L)**. Corresponding microscopic observation of ultrathin sections of callus (40× objective lens).

After inoculating *Acer truncatum* ‘Lihong’ stem segments onto the medium for 4 weeks, six types of calluses were observed. Type I appeared white and fluffy, exhibiting rapid growth and maintaining its original state without browning. Type II displayed white, granular characteristics with vigorous growth, often transforming to Type III and V calluses after successive generations. Type III appeared green and fluffy, rapidly growing with a less compact texture. Type IV manifested as green and compact, with slower growth and a tendency to brown and die in subsequent cultures. Type V appeared as vigorous, green granules, maintaining viability through successive generations. Type VI was identified as red granular and slow-growing, often transforming into Type IV callus in subsequent cultures (**Figures 3A–F**).

Microscopic examination of the six types of calluses after fixation revealed that the green granular callus comprised embryonic cell clusters with compact cell arrangement, small cell size, and centrally located nuclei. In contrast, the white granular callus contained embryonic cell clusters with irregular, large nucleated cells, indicating an intermediate state between embryonic and non-embryonic callus. Therefore, it is likely to transform into a green granular callus in subsequent generations. The remaining four types of calluses displayed large cell size, loose cell arrangement, and lacked obvious nucleus, indicative of non-embryonic callus (**Figures 3G–L**).

### Embryonic callus proliferation

**Figure 4:**
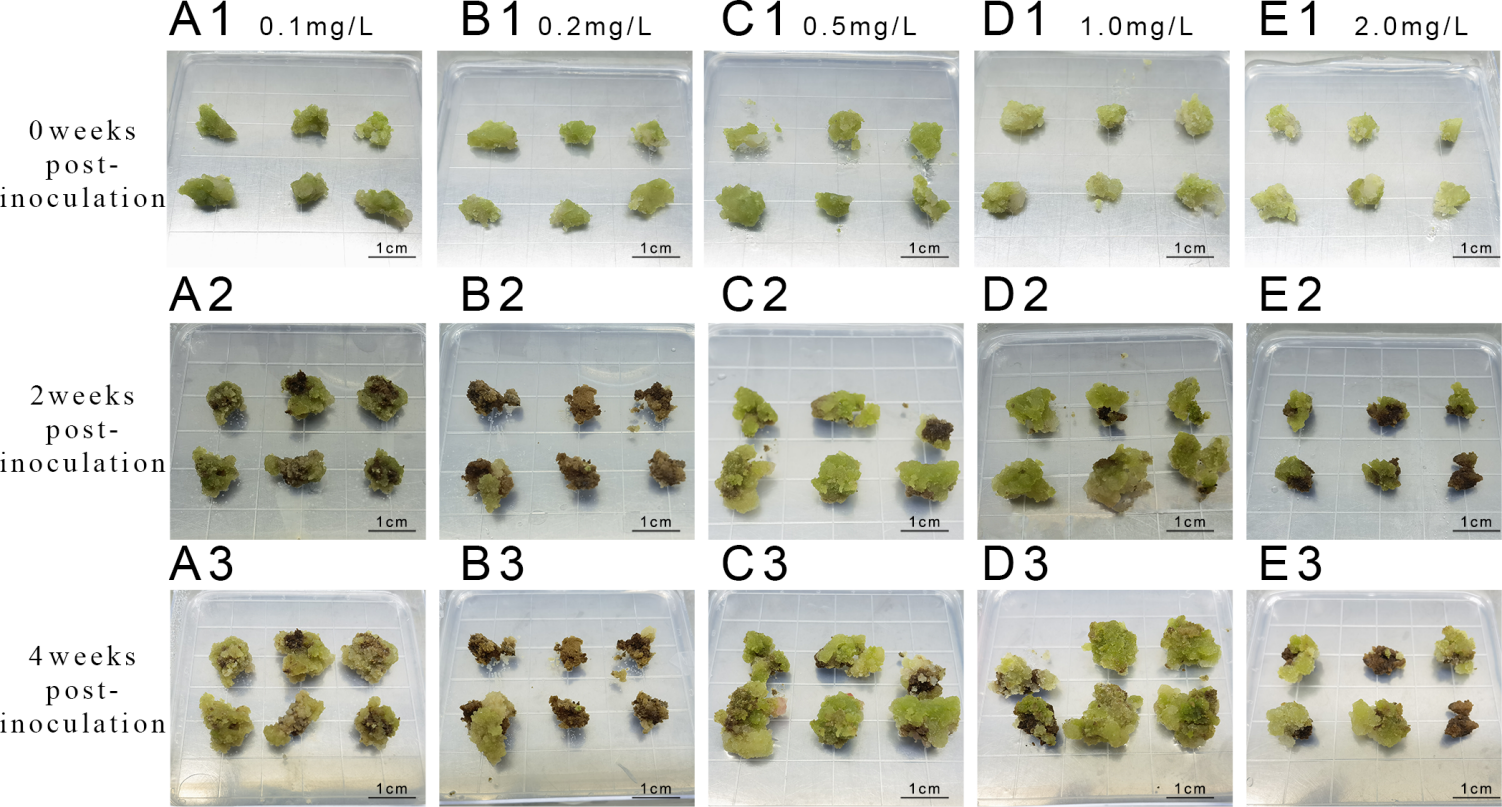
Proliferation of callus at different concentrations of KT **(A1-A3)** represent inoculation and culture with 0.1 mg/L KT; **(B1-B3)** represent inoculation and culture with 0.2 mg/L KT; **(C1-C3)** represent inoculation and culture with 0.5 mg/L KT; **(D1-D3)** represent inoculation and culture with 1 mg/L KT. **(E1-E3)** represent inoculation and culture with 2 mg/L KT.

Upon callus inoculation onto successive proliferation media containing varying concentrations of KT (0.1, 0.2, and 2.0 mg L^-1^) (**Figure 4**), changes were observed after 4 weeks of culture. The callus exhibited partial browning and poor growth, transitioning from a less compact yellowish-green to a dark green color, with increased browning and slower proliferation. Furthermore, the survival rate and proliferation coefficient of the callus in the medium with 1.0 mg L^-1^ KT were significantly higher than those in other treatment groups (P<0.05) (**Table 6**). The callus proliferation was rapid, displaying a less compact, granular morphology, indicating optimal growth conditions at 1.0 mg L^-1^ KT.

**Table 6:** Effect of different concentrations of KT on embryonic callus proliferation.

Medium No.	Plant Hormone (mg L^-1^)				Callus Condition			Biological Replicates
	TDZ	IBA	KT	GA_3_	Survival rate (%)	Browning/Mortality Rate (%)	Proliferation Coefficient	
1	1.0	0.5	0.1	0.1	52.36 ± 1.92d	47.64 ± 0.72a	1.53 ± 0.21bc	3
2	1.0	0.5	0.2	0.1	85.49 ± 0.78b	14.51 ± 0.71c	1.07 ± 0.18c	3
3	1.0	0.5	0.5	0.1	83.33 ± 1.22b	16.67 ± 0.95c	2.36 ± 0.14b	3
4	1.0	0.5	1.0	0.1	98.32 ± 0.74a	1.68 ± 0.38d	4.67 ± 0.7a	3
5	1.0	0.5	2.0	0.1	66.67 ± 1.48c	33.33 ± 1.36b	2.09 ± 0.36bc	3

Values in the same column followed by different lowercase letters indicate significant differences (P < 0.05).

**Figure 5:**
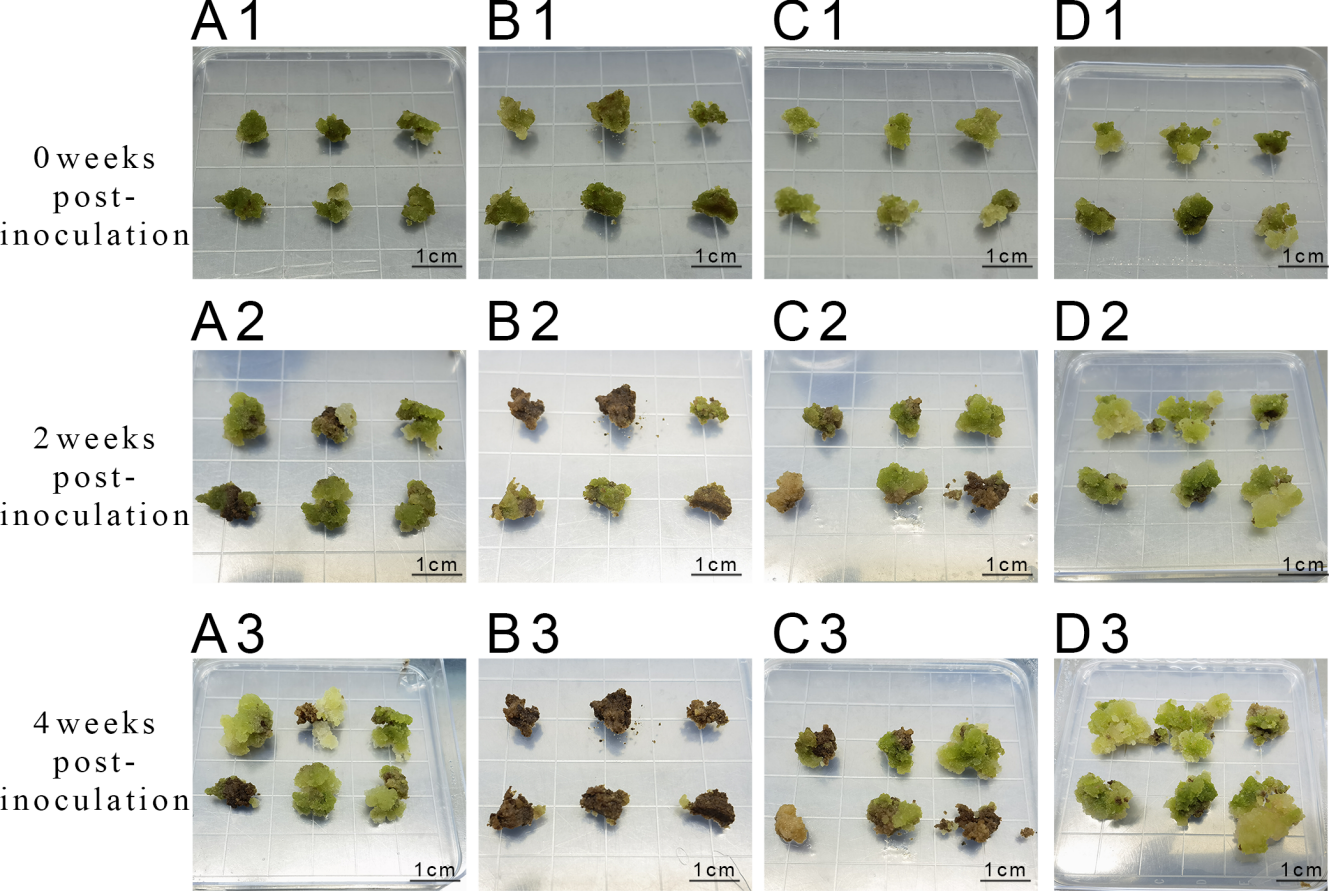
Proliferation of callus at different concentrations of 6-BA and KT **(A1-A3)** represent inoculation and culture with 2 mg/L 6-BA + 0.5 mg/L KT; **(B1-B3)** represent inoculation and culture with 2 mg/L 6-BA + 1.0 mg/L KT; **(C1-C3)** represent inoculation and culture with 3 mg/L 6-BA + 0.5 mg/L KT; **(D1-D3)** represent inoculation and culture with 3 mg/L 6-BA + 1.0 mg/L KT.

Exploration of the combined effects of 6-BA and KT (**Figure 5**) revealed robust callus growth on a medium containing 3 mg L^-1^ 6-BA + 1 mg L^-1^ KT after 4 weeks of incubation. The survival rate and proliferation coefficients were significantly higher than those of other treatment groups (**Table 7**).

**Table 7:** Effect of different combinations of KT and 6-BA concentrations on the embryonic callus proliferation.

Medium No.	Plant Hormone (mg L^-1^)					Callus Condition			Biological Replicates
	TDZ	IBA	KT	6-BA	GA_3_	Survival rate (%)	Browning/Mortality Rate (%)	Proliferation Coefficient	
1	1.0	0.5	0.5	2.0	0.1	15.23 ± 1.11d	84.77 ± 1.36a	0.51 ± 0.07c	3
2	1.0	0.5	0.5	3.0	0.1	65.29 ± 2.09c	34.71 ± 0.96b	1.08 ± 0.21bc	3
3	1.0	0.5	1.0	2.0	0.1	86.39 ± 0.79b	13.61 ± 1.12c	1.82 ± 0.11b	3
4	1.0	0.5	1.0	3.0	0.1	97.95 ± 1.17a	2.05 ± 0.40d	5.12 ± 0.49a	3

Values in the same column followed by different lowercase letters indicate significant differences (P < 0.05).

### Embryonic callus transformation

The gene overexpression vector pCAMBIA1305-*GST894*-GFP was introduced into *Agrobacterium* GV3101, which then infected the embryonic callus of *Acer truncatum* ‘Lihong’. During the inhibition culture, embryonic callus growth was monitored daily, and any heavily contaminated embryonic callus was promptly transferred to a new dish while assessing the inhibition efficiency. After the inhibition culture, the transformed embryonic callus was screened for resistance, determining the optimal screening concentration by testing various hygromycin gradients.

**Figure 6:**
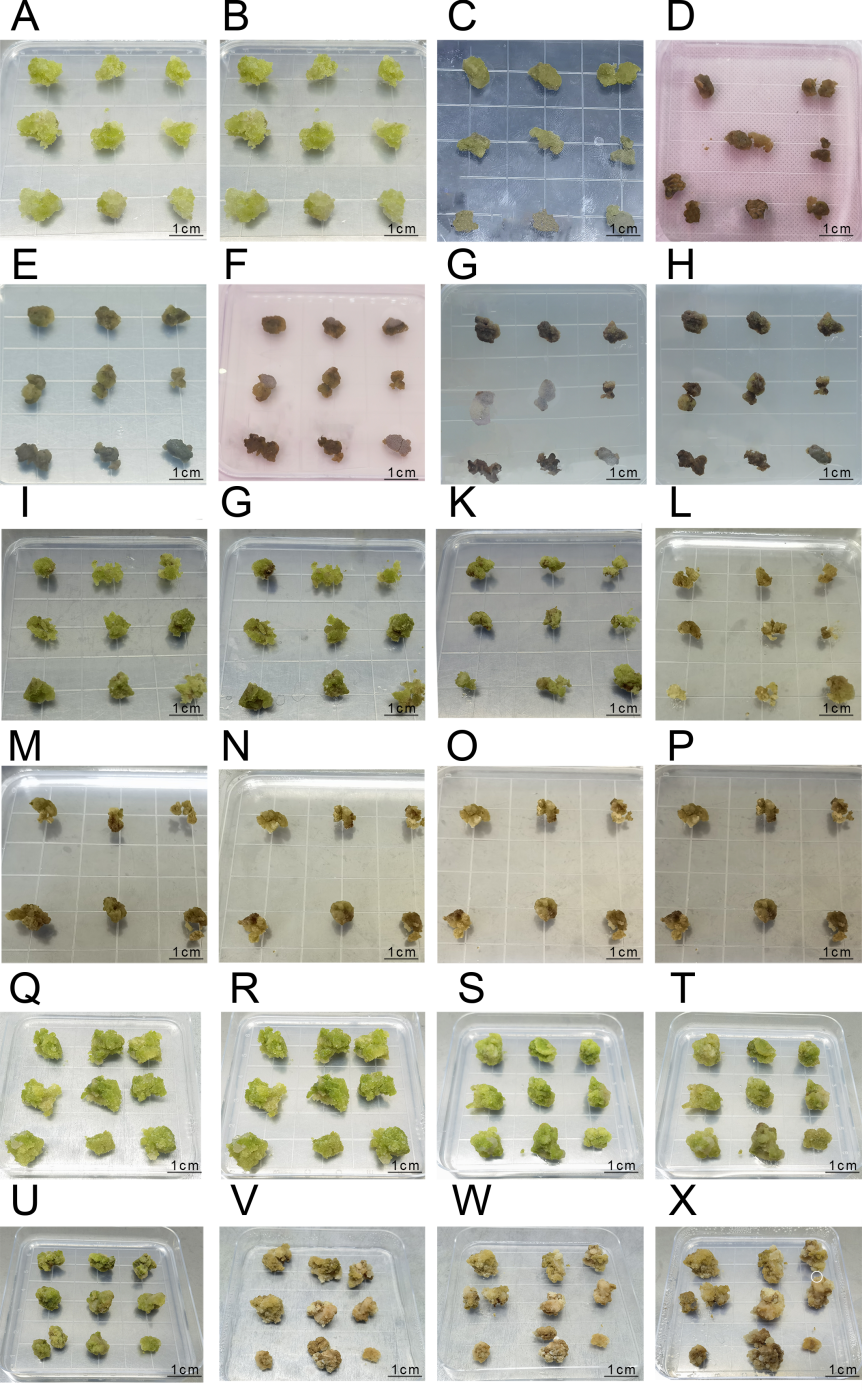
The Process of Agrobacterium Transformation in the First, Second, and Third Batches **(A-H)** represent the infection process of the first batch: **(A)** Pre-culture inoculation; **(B)** Pre-culture for 2 days; **(C)** Co-culture inoculation; **(D)** Co-culture for 60 hours; **(E)** Anti-bacterial culture inoculation; **(F)** Anti-bacterial culture for 1 day; **(G)** Anti-bacterial culture for 3 days; **(H)** Anti-bacterial culture for 5 days; **(I-P)** represent the infection process of the second batch:**(I)** Pre-culture inoculation; **(J)** Pre-culture for 2 days; **(K)** Co-culture inoculation; **(L)** Co-culture for 2 days; **(M)** Anti-bacterial culture inoculation; **(N)** Anti-bacterial culture for 1 day; **(O)** Anti-bacterial culture for 3 days; **(P)** Anti-bacterial culture for 5 days; **(Q-X)** represent the infection process of the third batch; **(Q)** Pre-culture inoculation; **(R)** Pre-culture for 2 days; **(S)** Co-culture inoculation; **(T)** Co-culture for 2 days; **(U)** Anti-bacterial culture inoculation; **(V)** Anti-bacterial culture for 15 days; **(W)** Selection culture inoculation; **(X)** Selection culture for 7 days.

In the first experimental batch (Sample A), slight browning was observed on the embryonic callus surface after 2 days of dark culture treatment. However, the overall condition remained satisfactory, with the color retaining a bright green or yellow-green hue. Upon *Agrobacterium* infestation, the embryonic callus initially exhibited no noticeable changes upon inoculation. However, after 60 hours of co-culture, significant surface browning occurred, accompanied by a marked reduction in vigor, indicating a substantial impact from *Agrobacterium* infestation. During inhibition culture, *Agrobacterium* gradually encapsulated the embryonic callus tissues from the third day onwards, culminating in complete browning and death by the fifth day (**Figures 6A–H**).

In the second experimental batch (Sample B), similar results were obtained, with slight browning observed on the embryonic callus surface after 2 days of dark culture treatment. However, the overall condition remained favorable, with the embryonic callus maintaining a bright green or yellow-green color. After 2 days of co-culture, noticeable surface browning occurred, accompanied by decreased vigor. Upon inhibition culture, *Agrobacterium* gradually encapsulated the embryonic callus tissues from the third day onwards, resulting in complete browning and death by the fifth day (**Figures 6I–P**).

In the third batch of experimental results, embryogenic callus tissue exhibited slight browning on the surface after 1 day of preculture treatment, but overall remained in good condition, with a bright green color (**Figures 6Q–R**). The embryogenic callus tissue was in good condition before co-cultivation after infection, and after 2 days of co-cultivation, obvious browning was observed, but the tissue still remained viable (**Figures 6S, T**). After co-cultivation with Agrobacterium, the embryonic callus tissue was inoculated onto an antibacterial medium. After 15 days of antibacterial culture, the embryonic callus tissue showed extensive browning, but small white granular embryonic callus appeared on the surface, indicating that the tissue still maintained some viability (**Figures 6U, V**).

After 7 days of culture on hygromycin-resistant selection medium following antibacterial culture, a small portion of the embryonic callus tissue completely browned and died. The remaining portion, although showing browning, still maintained viability and exhibited small white granular embryonic callus growth (marked with white circles in the image, **Figures 6W, X**), which can be used for subsequent PCR identification.

**Figure 7:**
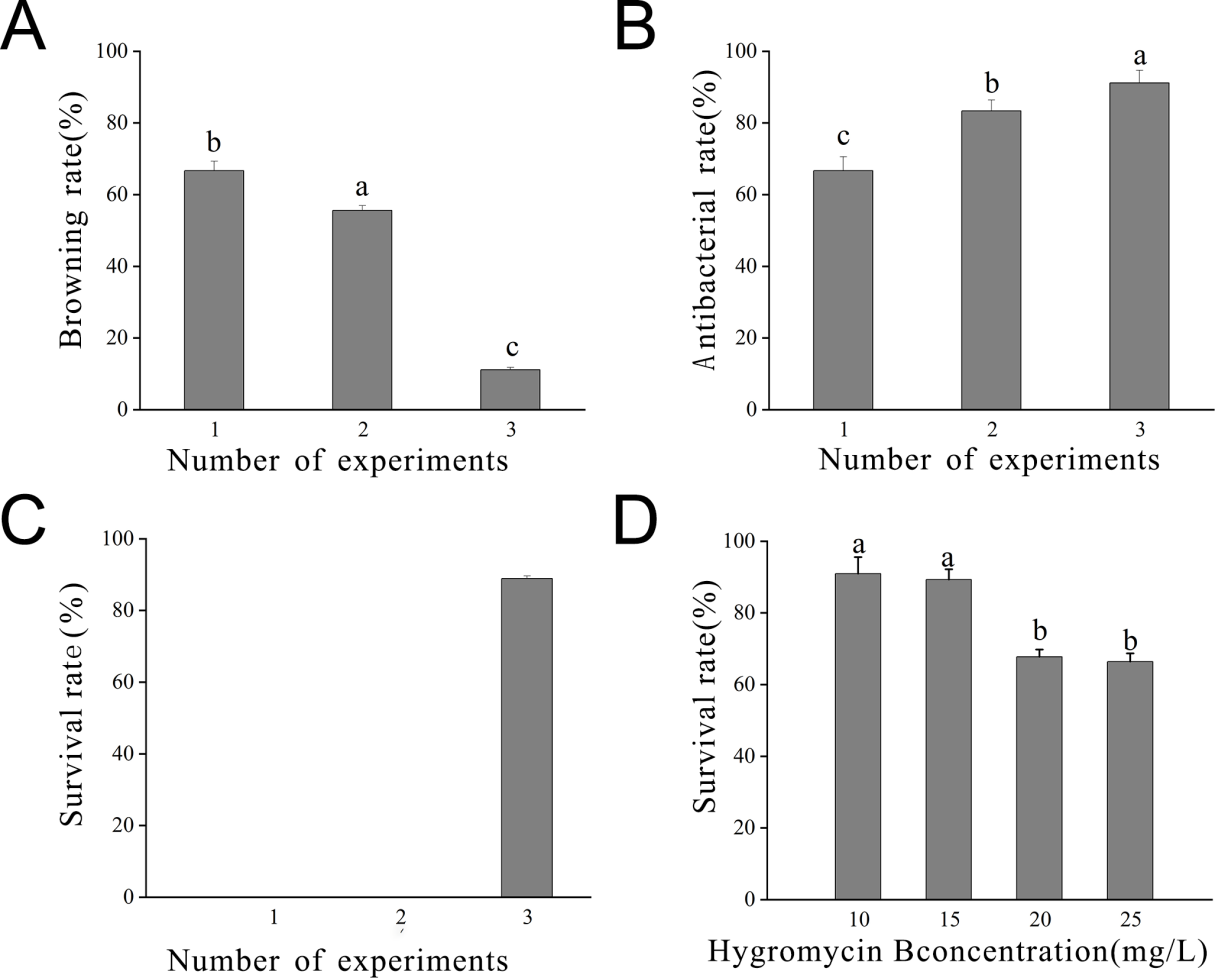
Statistics of embryonic callus transformation results **(A)** Browning rate observed after 7 days in the inhibition culture; **(B)** Inhibition rate observed after 7 days in the inhibition culture; **(C)** Survival rate observed after 7 days in the inhibition culture; **(D)** Survival rate observed at different concentrations of hygromycin. Lowercase letters indicate significant differences among measurement data from different experimental batches at the *P* < 0.05 level. The data represent the mean of three biological replicates.

Statistical analysis was conducted on bacterial inhibition post-callus infestation. Across the three infestation trials, the browning rate gradually decreased due to the change in the medium. Notably, the browning rate in the third batch was significantly lower than in the initial two batches (P < 0.05). Concurrently, the inhibition rate gradually increased, indicating that the embryonic callus differentiation medium promoted embryonic callus growth, thereby enhancing resistance to *Agrobacterium* infestation. This resulted in an elevated inhibition rate and viability (**Figure 7**).

**Figure 8:**
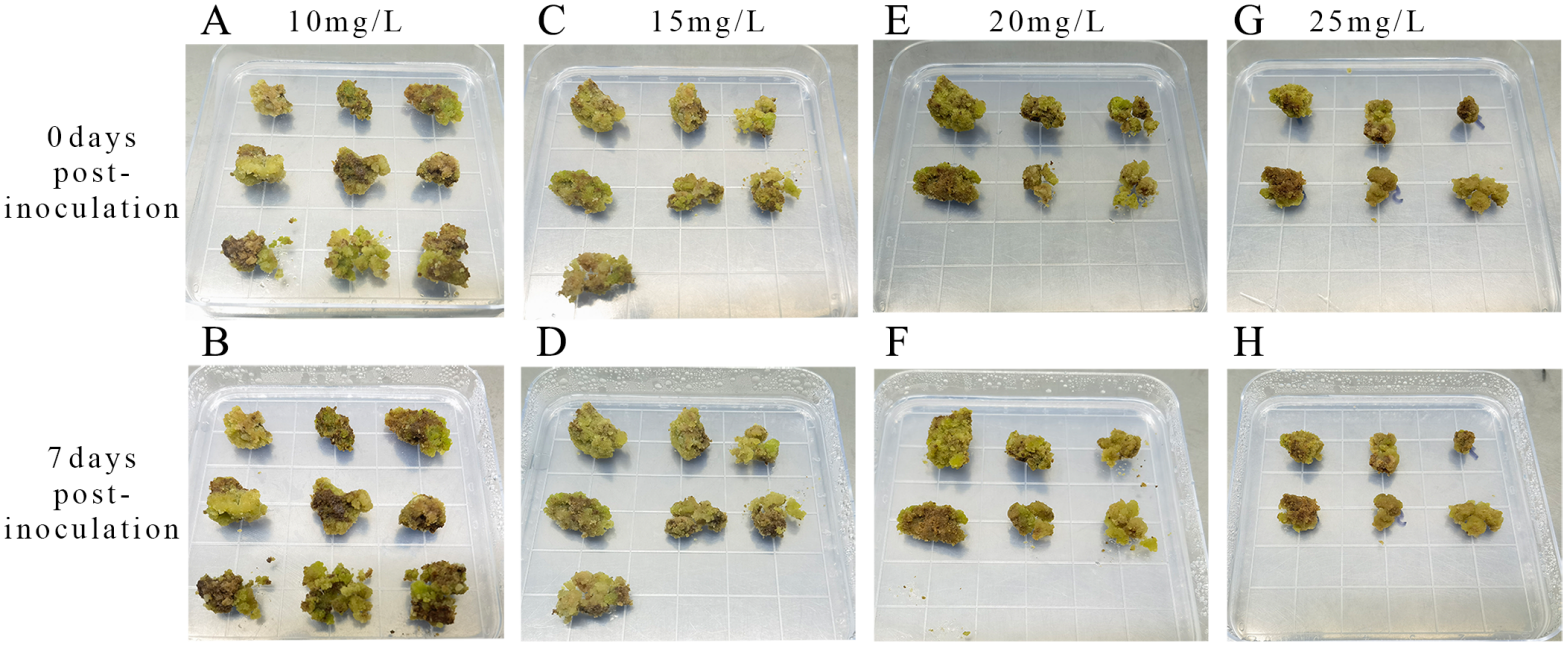
Screening of hygromycin concentrations **(A)** Inoculation with 10 mg L^-1^ hygromycin; **(B)** 7 days culture with 10 mg L^-1^ hygromycin; **(C)** Inoculation with 15 mg L^-1^ hygromycin; **(D)** 7 days culture with 15 mg L^-1^ hygromycin; **(E)** Inoculation with 20 mg L^-1^ hygromycin; **(F)** 7 days culture with 20 mg L^-1^ hygromycin; **(G)** Inoculation with 25 mg L^-1^ hygromycin; **(H)** 7 days culture with 25 mg L^-1^ hygromycin.

The optimal hygromycin concentration was determined through screening. Initially, the embryonic callus exhibited no significant changes when grown on a medium containing 10 mg L^-1^ of hygromycin for 7 days. However, with increasing hygromycin concentration, the embryonic callus tissues started browning and deteriorating, indicating unsuccessful infestation and inability to withstand hygromycin stress (**Figure 8**). Moreover, one-way ANOVA showed that the embryonic callus survival rate on media containing 20 mg L^-1^ and 25 mg L^-1^ hygromycin was significantly lower than in other treatments (P < 0.05), yet it remained above 60%. This suggests that 25 mg L^-1^ hygromycin is more suitable for screening transgenic embryonic callus of *Acer truncatum* ‘Lihong’ (**Figure 7D**).

### Identification of transgenic positive embryonic callus

**Figure 9:**
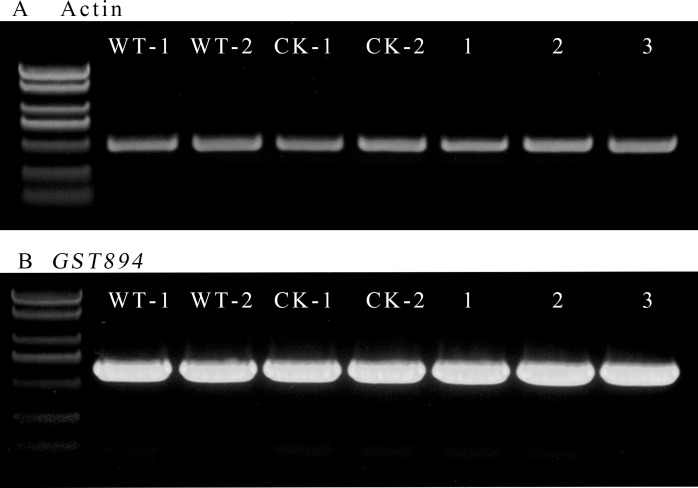
RT-PCR electropherogram **(A)** Amplification bands of the internal control gene **(B)** Amplification bands of the *GST894* gene; WT-1 and WY-2 represent the untransformed embryonic callus tissues; CK-1 and CK-2 represent the embryonic callus tissues transformed with the p1305.1 empty vector; 1-3 represent the embryonic callus tissues transformed with *GST894*.

Select five pieces of well-growing, non-browned transgenic embryonic callus tissue after selection as experimental materials. Use uninfected embryonic callus tissue as a control for RNA extraction. Verify the quality of the extracted RNA via agarose gel electrophoresis. Then, measure the RNA purity and concentration with a microvolume spectrophotometer, ensuring they meet the required standards. Using the extracted RNA, reverse transcribe to obtain cDNA, which will serve as the template. Design primers for the Acer truncatum actin gene, and perform gene cloning using Fast Taq DNA Polymerase. Confirm the expected length of the amplified products via gel electrophoresis. Adjust the amount of template used in the amplification multiple times until the intensity of the target bands is consistent across samples (**Figure 9**).

**Figure 10:**
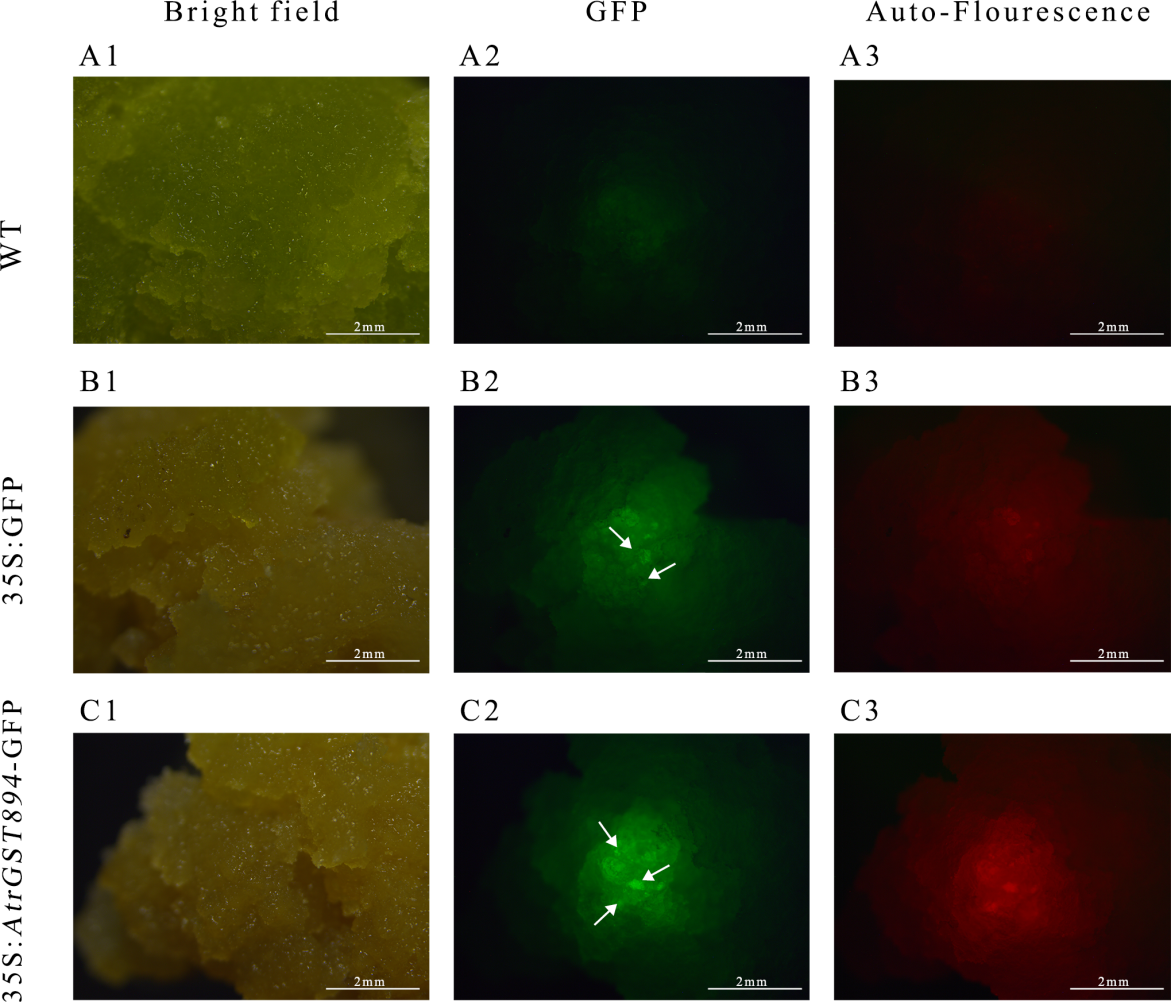
GFP fluorescence detection image. **(A1)** Bright field of non-transformed callus **(A2)** GFP fluorescence of non-transformed callus **(A3)** Autofluorescence of non-transformed callus **(B1)** Bright field of transformed empty vector callus **(B2)** GFP fluorescence of transformed empty vector callus **(B3)** Autofluorescence of transformed empty vector callus **(C1)** GFP fluorescence of transformed target gene callus **(C2)** Bright field of transformed target gene callus **(C3)** Autofluorescence of transformed target gene callus.

Use the same amount of template used for Actin amplification and, with the designed *GST894* primers, perform gene cloning using Fast Taq DNA Polymerase. Gel electrophoresis shows that the *GST894* gene is overexpressed after transformation (**Figure 9**). Conduct GFP fluorescence detection on the embryonic callus tissues. Significant fluorescence signals can be observed in the empty vector and *GST894*-transformed embryonic callus tissues (indicated by white arrows in the figure), while no fluorescence signal is detected in the untransformed embryonic callus tissues (**Figure 10**).

### Expression of anthocyanin pathway-related genes in transgenic embryonic callus tissues

**Figure 11:**
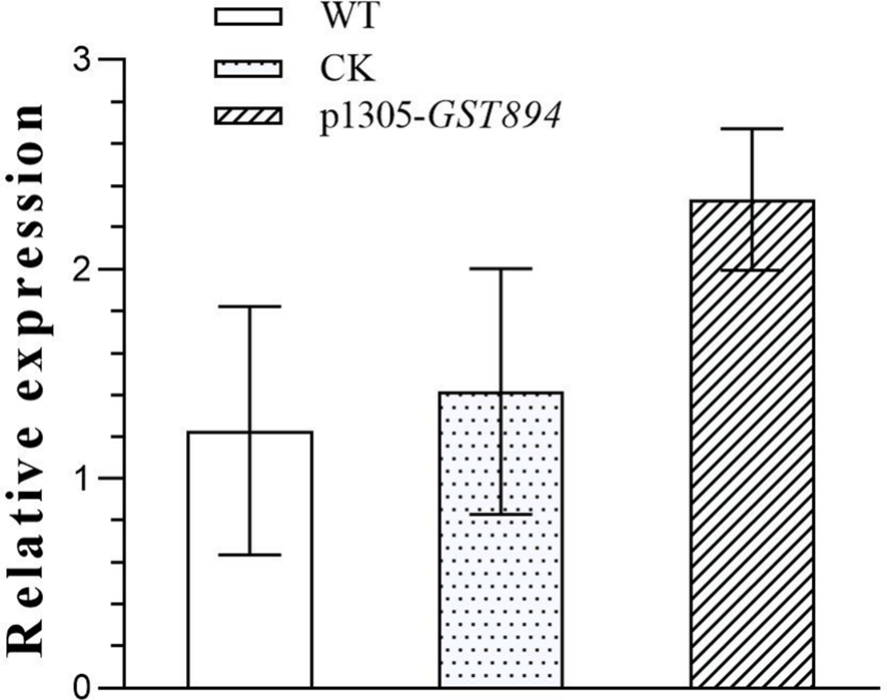
Relative Expression of *GST894* Gene in Embryogenic Callus Tissues.

Perform qRT-PCR detection on uninfected embryonic callus tissues, embryonic callus tissues transformed with the empty vector, and embryonic callus tissues transformed with the *GST894* gene. The results show that the gene expression level in the *GST894*-transformed embryonic callus tissues is significantly higher than in the uninfected callus tissues and the embryonic callus tissues transformed with the empty vector. This indicates that the *GST894* gene is overexpressed in the *GST894*-transformed embryonic callus tissues (**Figure 11**).

## Discussion

Outdoor-collected plant materials harbor more bacteria and fungi due to natural growth conditions, making cleaning and sterilization challenging. Prolonged sterilization risks plant death, whereas shorter durations increase contamination rates during subsequent cultures. *Acer truncatum* ‘Lihong’ produces abundant phenolics as a defense mechanism against insect bites; however, phenolics aggravate callus browning. The experiment showed significantly higher browning rates in callus induced from outdoor materials than in sterile seedlings. Additionally, outdoor material collection is time-sensitive, requiring the collection of new spring shoots. Therefore, sterile seedlings grown indoors were selected as test materials for this study.

Compared to leaves, stem segments exhibit less differentiation, which explains their rapid callus induction capability. Sterile seedlings demonstrate a higher callus emergence rate compared to new shoots of outdoor plants. In this study, callus appeared at the cut ends of the stem segments as early as 3 days. The length of stem segment inoculation also influenced callus induction, with the optimal length being 1 cm. Unlike most plants, *Acer truncatum* ‘Lihong’ callus induction conditions require a 16-h/8-h (light/dark)culture cycle. For instance, more than 20 days of dark culture was required to induce callus in the young stem of *Fraxinus velutina* Torr. (Zong, 2003), and approximately 20 days of dark culture was required to induce callus in the mature embryo of *Ginkgo biloba* L (Mu et al., 2014), suggesting a preference for light in *Acer truncatum* ‘Lihong’.

Numerous endogenous hormones naturally occur in plants, regulating plant growth and development. The concentration ratio of exogenous growth hormones and cytokinins influences the induction and differentiation of *Acer truncatum* ‘Lihong’ callus. In particular, 2,4-D is the hormone most effective for callus induction, leading to high-quality callus formation, typically appearing as yellow-green or white crystal-clear granules.

Both 6-BA and NAA also positively affect callus induction. Newly induced callus from stem segments and leaves is predominantly white or green. However, without continuous succession culture, the callus gradually yellows, eventually forming a fluffy mass until it dies from browning. Therefore, an uninterrupted succession culture, ideally every 2-4, is necessary. Stable proliferation is achieved by transferring the callus to a fresh medium. Six callus types emerge after successive cultures: white fluffy, white granular, green fluffy, green compact, green granular, and red callus. During succession, callus should be cut into soybean-sized pieces; excessively small callus may die due to the lack of a population effect. Green granular callus can be initially identified as embryonic callus by making ultrathin sections of each type and observing the cell clusters.

WPM, developed by Lloyd and McCown in 1981 for *Kalmia latifolia* stem tip culture, is a modification of MS medium. Compared to MS, WPM contains one-fourth of the ammonium nitrate and supplies nitrogen salts in the form of calcium nitrate. Currently, low-salt WPM is commonly used for woody plants. Li discovered that WPM served as a suitable basal medium for Yunnan golden tea tissue culture, with optimal proliferation achieved using WPM + 3.0 mg L^-1^ 6-BA + 0.30 mg L^-1^ IAA, resulting in a proliferation coefficient of 6.83 (Li, 2018). In *Camellia oleifera* Abel young embryo culture, Xiaoming found success with WPM + 2.0 mg L^-1^ 6-BA + 1.5 mg L^-1^ NAA for seedling growth (success rate of 90.00%) and WPM + 1.0 mg L^-1^ 6-BA + 0.5 mg L^-1^ NAA for embryonic callus induction from immature cotyledons (Fan, 2011). Therefore, in this experiment, WPM served as the basal medium for callus proliferation and differentiation stages, supplemented with appropriate plant growth regulators to achieve rapid, healthy callus proliferation and clear differentiation trends.

Plant growth regulators play a vital role in regulating cell differentiation and organogenesis. Research has shown that growth media containing cytokinins promote shoot formation in micropropagation, with a higher ratio of cytokinins to auxins required for shoot regeneration. Studies have also demonstrated the induction of adventitious buds directly from the flowering stems of *Iris tectorum* Maxim. using various cytokinins, such as KT and 6-BA (Liu et al., 2020). In this study, different concentrations of KT significantly influenced callus proliferation. With increasing KT concentration, the callus exhibited robust growth followed by gradual browning, and the proliferation rate initially increased before decreasing, indicating the significant impact of KT on improving proliferation. Furthermore, combining KT with the later addition of 6-BA further improved the proliferation effect. Although higher concentrations of cytokinins could negatively affect callus proliferation, the synergistic effect of different cytokinins effectively enhanced proliferation.

During embryonic callus proliferation culture, performing successive selection is essential to ensure a homogenous, less compact embryonic callus. Failure to do so may result in a mixture of embryonic and non-embryonic callus, as well as variations in callus texture. During proliferation, embryonic callus degradation is inevitable, and some may lose their embryonic nature due to the competitive advantage of non-embryonic cells. Min et al. emphasized the dominance of non-embryonic cells in growth and the importance of early screening to eliminate them, maintaining inoculum uniformity (Min, 2007). Additionally, the duration of succession culture affects callus quality. A prolonged period may lead to loss of embryonic characteristics due to nutrient deficiency, while a short period increases workload and causes mechanical damage, hindering callus growth.

In this experiment, the induction process of *Acer truncatum* ‘Lihong’ embryonic callus was initially developed. However, after approximately 4 months of continuous succession, the callus ceased growth, turned brown, and eventually perished. This suggests that the callus obtained did not develop into an embryonic callus capable of differentiation. Furthermore, the re-differentiation step to form adventitious buds was not achieved, possibly indicating a need for additional external stimuli in addition to hormonal induction for shoot regeneration. Therefore, further investigation into embryo induction methods remains crucial for future research.

We chose to use Agrobacterium tumefaciens to infect the embryogenic callus of *Acer truncatum*. At present, the embryogenic callus is growing continuously, and the browning of the embryogenic callus should be paid attention to continuously in the future. When the embryogenic callus is without browning and growing well is screened for subsequent transgenic callus. The embryogenic callus was used as the infection material to verify that we successfully transferred the target gene into the embryogenic callus, which means that the genetic transformation system of *Acer truncatum* embryogenic callus was partially completed, and the infection process could be effectively inhibited. Although there was partial browning, it could continue to proliferate. Therefore, in future experiments, the focus is still to continue to verify the optimal conditions for optimizing the genetic transformation of *Acer truncatum* embryogenic callus and to solve the problems of difficulty in embryonic callus germination.

## Data Availability

The data presented in the study are deposited in the NGDC repository, accession number PRJCA014724.

## Supplementary material

Supplementary Figure 1 The five stages of leaf color change in the two varieties

Supplementary Figure 2 Cultivation of *Acer truncatum* ‘Lihong’ sterile seedlings **(A)** *Acer truncatum* ‘Lihong’ seeds; **(B)** *Acer truncatum* ‘Lihong’ seeds inoculated for 1 week; **(C)** 3-week-old *Acer truncatum* ‘Lihong’ sterile seedlings; **(D)** 7-week-old *Acer truncatum* ‘Lihong’ sterile seedlings.

Supplementary Figure 3 The vector map of pCAMBIA1305.1-GFP.

Supplementary Figure 4 GO function classification diagram of unigene

Supplementary Figure 5 Venn diagram of differential genes

Supplementary Figure 6 The expression analysis of key enzymes and transporter genes in the anthocyanidin biosynthesis pathway

Supplementary Figure 7 qRT-PCR of the Key Candidate Gene *GST894* in the Anthocyanin Metabolic Pathway
