# Supplementary material for: Preliminary establishment of genetic transformation system for embryogenic callus of Acer truncatum ‘Lihong’
Source: Front Plant Sci. 2024 Sep 5;15:1419313. doi: 10.3389/fpls.2024.1419313 (PMC11410635; doi:10.3389/fpls.2024.1419313)
Supplement: Supplementary Figure 1 — The five stages of leaf color change in the two varieties [file DataSheet1.docx]

Supplementary Material

Preliminary establishment of genetic transformation system for embryogenic callus of Acer truncatum Bunge

Yipeng Yang^1†^, Yuan Chan^2^ ^†^,Yongge Wang^1 †^, Hao guo^2^, Lina Song^1^, Huali Zhang^1^, Liping Sun^1^, Richen Cong^1^ and Hua Zhang ^1*^

*** Correspondence:** Hua Zhang: [seastory@163.com](mailto:seastory@163.com)

# Supplementary Supporting article

1. Induction and Proliferation of Loose Embryogenic Callus in Euonymus maackii;

**The original paragraph :**

2. 2 不同植物激素浓度配比对胚性愈伤组织形成的影响

由表 3 可知，不同培养基上愈伤组织胚性情况、愈伤组织特性与生长速度存在较大差异。当继续在松散型愈伤组织诱导培养基上进行培养，即只附加 1.0 mg /L 2，4－D 时，愈伤组织保持较快速度的增殖生长，为结构松散、质地较硬的白色愈伤组织，但显微镜观察发现此类愈伤组织未能被醋酸洋红染色，且细胞较大，细胞质浓度低、液泡较大，为典型的非胚性愈伤组织（图 2A）。在培养基中只附加一定浓度的 BA 时，愈伤组织质地坚硬，结构紧密，颜色逐渐变绿，生长速度缓慢，显微镜观察发现此类愈伤组织多数内部组织化且分化出了厚壁组织、导管等，此类愈伤组织已经失去薄壁细胞的特点，分化为成熟组织，仅愈伤组织表层有少量分生组织细胞。当培养基中 BA 和 2，4－D 的比值较高时，即 MS+1.0 mg /L BA+0.5 mg /L 2，4－D 时，愈伤组织结构紧密、质地较硬，仅愈伤组织部分边缘细胞可被醋酸洋红染色，染色的细胞体积小、细胞核大、胞质浓，表现为胚性细胞特性（图 2B）;而当培养基中 BA 和 2，4－D 的比值较低时，即 MS+0.5 mg /L BA+1. 0 mg /L 2，4－D，愈伤组织结构松散、质地较硬，仅少数细胞可被醋酸洋红染色，表现为胚性细胞特性。当培养基中 BA 和 2，4－D 的比值一致时，即 MS+0.5 mg /L BA+0.5 mg /L 2，4－D上，愈伤组织能够被醋酸洋红溶液染为红色，为典型的胚性愈伤组织特征，且此时愈伤组织结构松散、质地较硬 ( 图 2D) ，生长速度也较快。因此，MS+0.5 mg /L BA+0.5 mg /L 2，4－D 为丝棉木松散型胚性愈伤组织诱导的理想培养基。

**Translation:**

2.2 Effects of different plant hormone concentration ratios on the formation of embryogenic callus

Table 3 shows that there are significant differences in embryonic conditions, callus characteristics, and growth rates of callus tissues on different media. When cultured on a loose callus induction medium supplemented with only 1.0 mg/L 2,4-D, the callus tissues proliferated at a fast rate, forming white callus with a loose structure and hard texture. However, microscopic observation revealed that this type of callus tissue could not be stained by acetocarmine, had larger cells with low cytoplasm density and large vacuoles, and was typical of non-embryonic callus tissue (Figure 2A).When the medium was supplemented with a certain concentration of BA, the callus tissue became hard, compact in structure, and gradually turned green, with a slow growth rate. Microscopic observation showed that most of these callus tissues were internally organized and differentiated into sclerenchyma tissues, ducts, etc., losing the characteristics of parenchyma cells and differentiating into mature tissues, with only a small number of meristematic cells on the surface of the callus tissue.When the ratio of BA to 2,4-D in the medium was high, i.e., MS + 1.0 mg/L BA + 0.5 mg/L 2,4-D, the callus tissue was compact in structure and hard in texture, with only some edge cells of the callus tissue being stained by acetocarmine. These stained cells were small, with large nuclei and dense cytoplasm, showing characteristics of embryonic cells (Figure 2B). When the ratio of BA to 2,4-D in the medium was low, i.e., MS + 0.5 mg/L BA + 1.0 mg/L 2,4-D, the callus tissue was loose in structure and hard in texture, with only a few cells stained by acetocarmine, exhibiting embryonic cell characteristics.When the ratio of BA to 2,4-D in the medium was equal, i.e., MS + 0.5 mg/L BA + 0.5 mg/L 2,4-D, the callus tissue could be stained red by acetocarmine solution, showing typical embryonic callus tissue characteristics. At this time, the callus tissue had a loose structure and hard texture (Figure 2D), with a fast growth rate. Therefore, MS + 0.5 mg/L BA + 0.5 mg/L 2,4-D is the ideal medium for inducing loose embryonic callus tissue in Euonymus alatus.


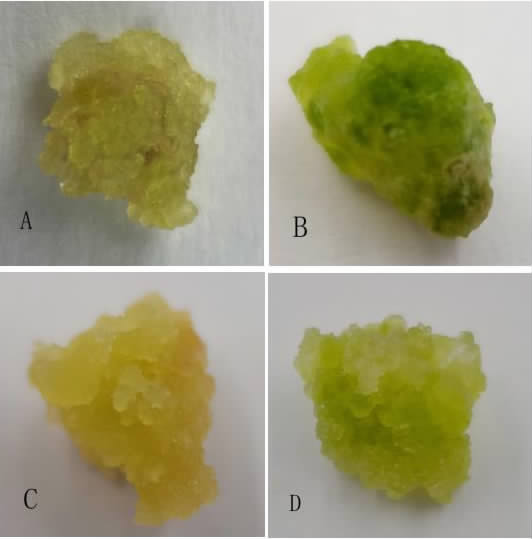


Fig. 1 Different types of callus

Note : A is semi-transparent callus with loose structure and soft texture ; B is callus with compact structure and hard texture ; C is callus with loose structure and hard texture ; D is embryonic callus with loose structure and hard texture.


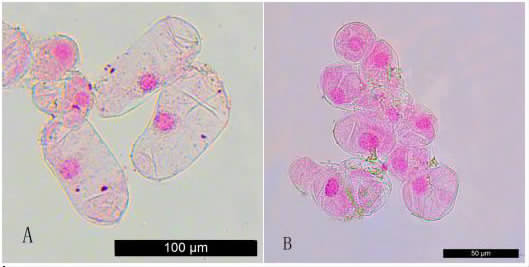


Fig. 2 Microscopic observation of callus

Note : A is non-embryogenic callus, B is embryonic callus.

1. Molecular cloning and characterization of fve genes from embryogenic callus in Miscanthus lutarioriparius;

Paraffin sections in the figure showed that there was obvious separation between the cell clusters of embryogenic callus, but no separation between the cell clusters of non-embryogenic callus.


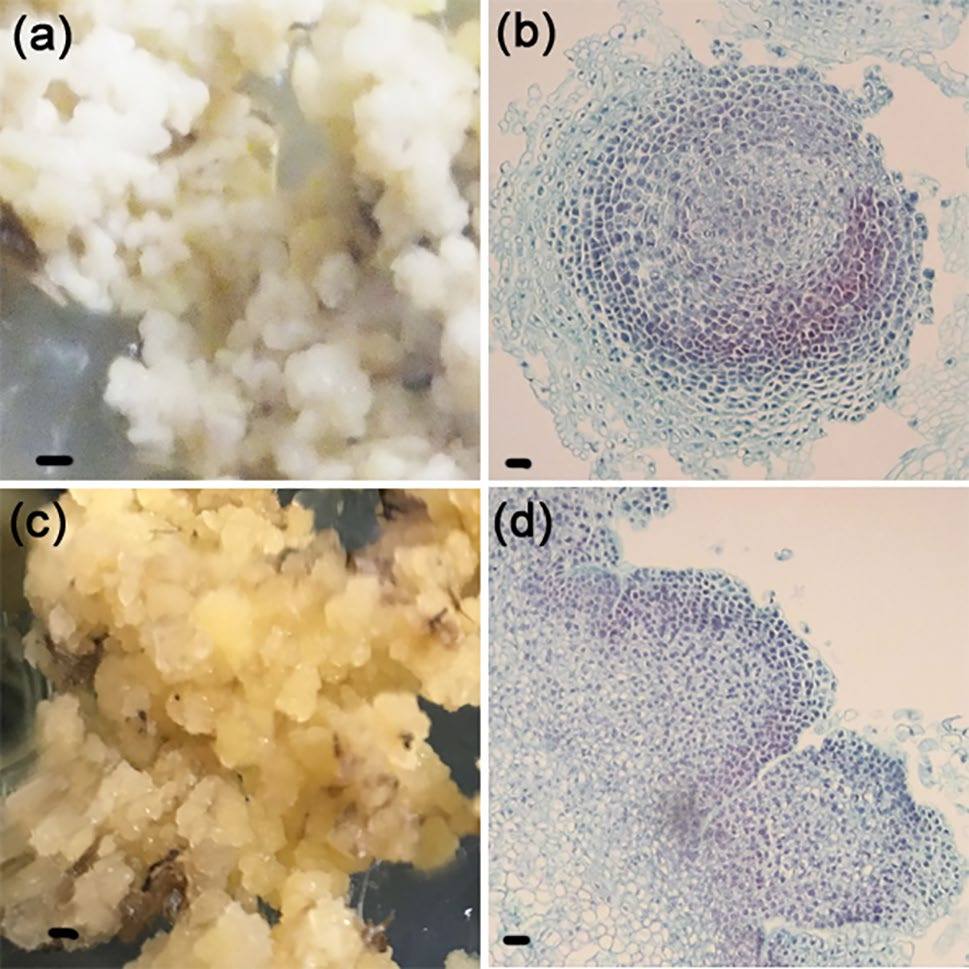


Fig. 1 Two types of callus and their parafn section, optical microscope (10×100).

a Embryogenic callus, b embryogenic callus paraffn section, c non-embryogenic callus, and d nonembryogenic callus parafn section

1. Culture System on Loose，Homogeneous，Embryogenic Callus of Populus×canadensis Moench‘Tower’;

**The original paragraph :**

2.1激素、无机盐和蔗糖对塔杨松散型愈伤组织 LC的诱导

表1的结果显示，用MS培养基培养时(蔗糖 浓度30 g·L^-1^)，选用BA、KT、2，4-D等激素的各种浓度组合都不能诱导出LC(表1: 配方1-1～1-13)，诱导出的都是绿色、紧密型的愈伤组织。这说明在诱导LC过程中激素不是关键因素。当仅仅降低培养基中无机盐含量到1/4MS或仅仅 降低蔗糖的含量至10 g·L^-1^ 时，也不能诱导出LC (表1:配方1-14～1-17)。只有当蔗糖浓度降 低至10 g·L^-1^ 和无机盐含量降低到原浓度的1/4 时(1/4MS)才能获得LC，获得率为85%～100% (表1: 配方1-18～1-22)。说明培养基的渗透压对形成愈伤的种类影响较大。可能是影响到了愈伤组织的结构，如MS培养基或高蔗糖(30 g·L^-1^)培养基都诱导出的是紧密型愈伤组织。 这种紧密型愈伤组织细胞排列紧密，细胞间隙较小，组织中细胞大小不一，并分化出导管等细胞，外观深绿色，是一些质地十分坚硬的大块状愈伤组织(图1)。这类组织很难分化出胚性愈伤和很难诱导出体细胞胚。而降低渗透压后形成的LC 细胞较小，多为圆球形，排列疏松，胞间隙明显;外 观为淡黄色或黄绿色，组织质地松软(图2)。这类组织容易分化出胚性愈伤组织。

**Translation:**

2.1 Effects of hormones, inorganic salts and sucrose on the induction of LC from loose callus of Populus cathayana

The results in Table 1 show that when cultured on MS medium with a sucrose concentration of 30 g·L-1, various combinations of hormones such as BA, KT, and 2,4-D could not induce the formation of loose callus (LC) (Table 1: Formulations 1-1 to 1-13). Instead, they induced the formation of green, compact callus tissues. This indicates that hormones are not the key factor in inducing LC. Lowering the inorganic salt content in the medium to 1/4 MS or reducing the sucrose content to 10 g·L-1 alone also did not induce LC (Table 1: Formulations 1-14 to 1-17). LC could only be obtained when the sucrose concentration was reduced to 10 g·L-1 and the inorganic salt content was reduced to 1/4 of the original concentration (1/4 MS), with an induction rate of 85% to 100% (Table 1: Formulations 1-18 to 1-22). This indicates that the osmotic pressure of the medium significantly affects the type of callus formed. High osmotic pressure, such as that in MS medium or high-sucrose (30 g·L-1) medium, induces the formation of compact callus tissue. These compact callus tissues have tightly arranged cells with small intercellular spaces, cells of varying sizes, and differentiated duct cells. They appear dark green and are very hard and blocky (Figure 1). Such tissues are difficult to differentiate into embryonic callus and are challenging for somatic embryogenesis. In contrast, LC formed under reduced osmotic pressure has smaller, mostly spherical cells arranged loosely with obvious intercellular spaces. It appears light yellow or yellow-green, and the tissue is soft (Figure 2). These tissues are more likely to differentiate into embryonic callus tissues.


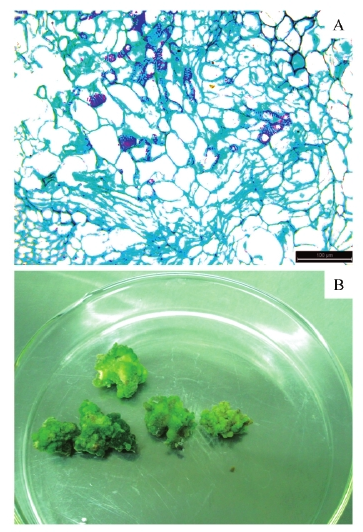


Fig．1 The compact callus microstructure and mor phology

1. Micrograph(10×40);B．Appearance


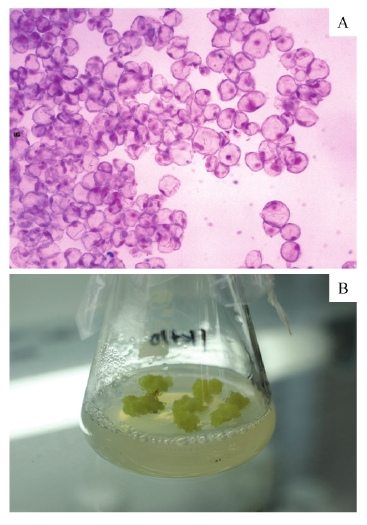


Fig．2 The loose callus microstructure and morphology

A．Micrograph(10×40);B．Appearance
