# Supplementary figures and images for: Preliminary establishment of genetic transformation system for embryogenic callus of Acer truncatum ‘Lihong’
Source: Front Plant Sci. 2024 Sep 5;15:1419313. doi: 10.3389/fpls.2024.1419313 (PMC11410635; doi:10.3389/fpls.2024.1419313)

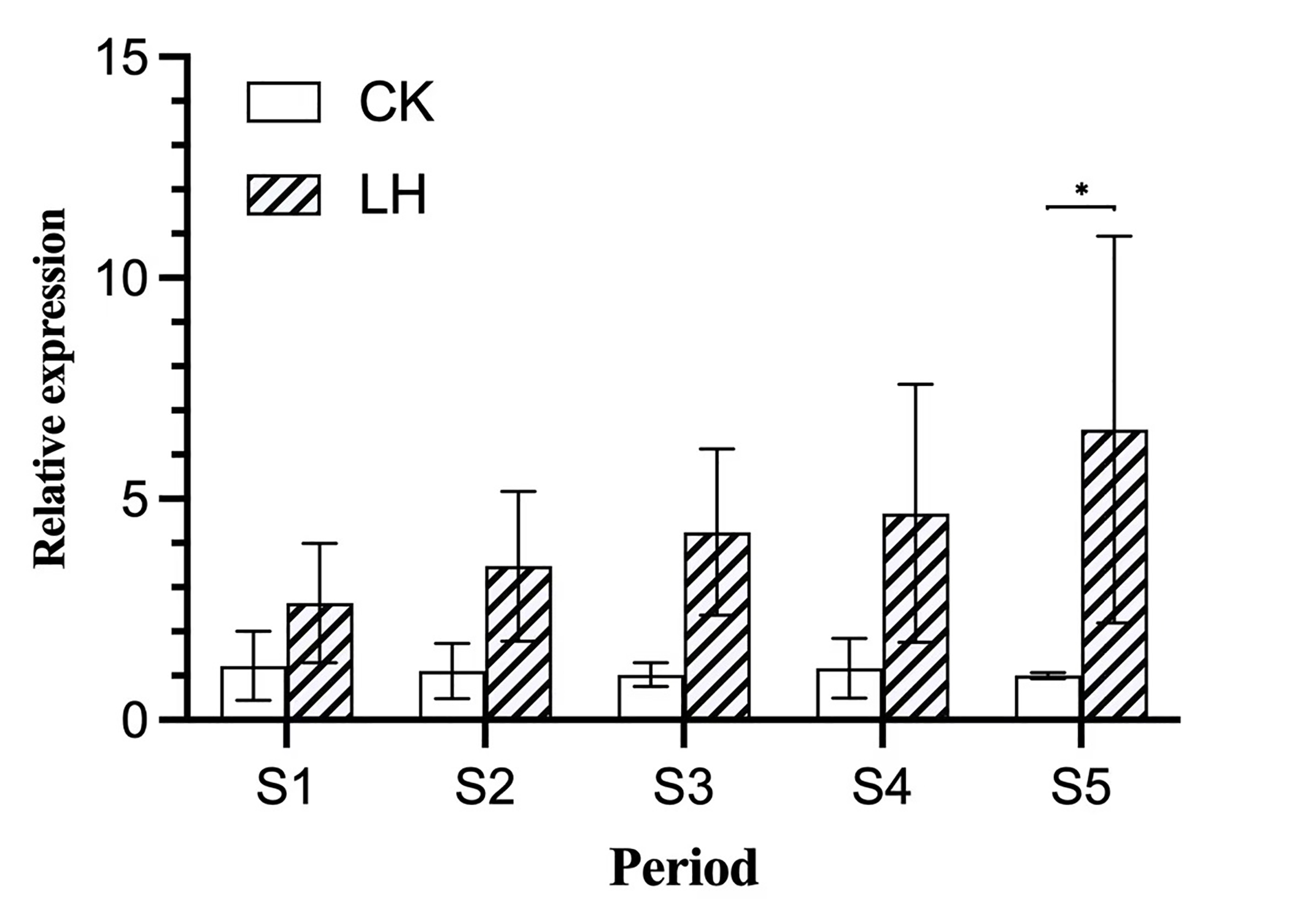

Supplement: Supplementary file 2 [file DataSheet2.zip › Supplementary Figure7.tif]

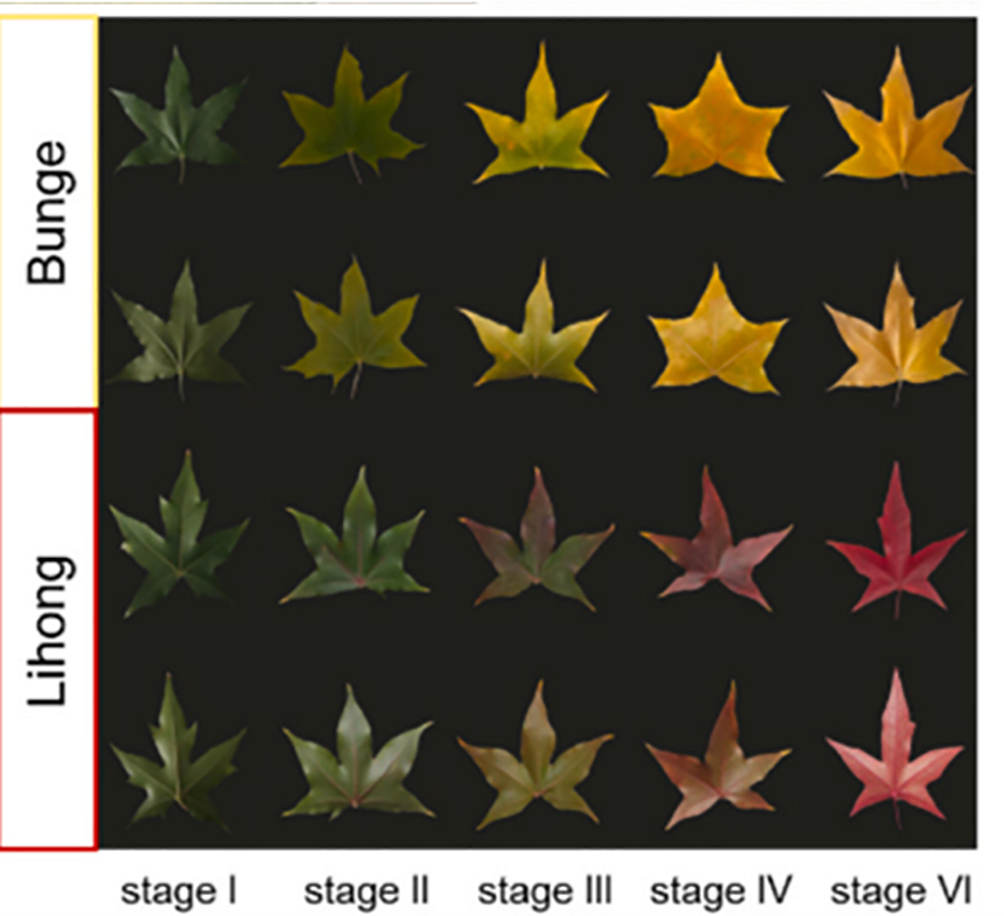

Supplement: Supplementary file 2 [file DataSheet2.zip › Supplementary Figure1.tif]

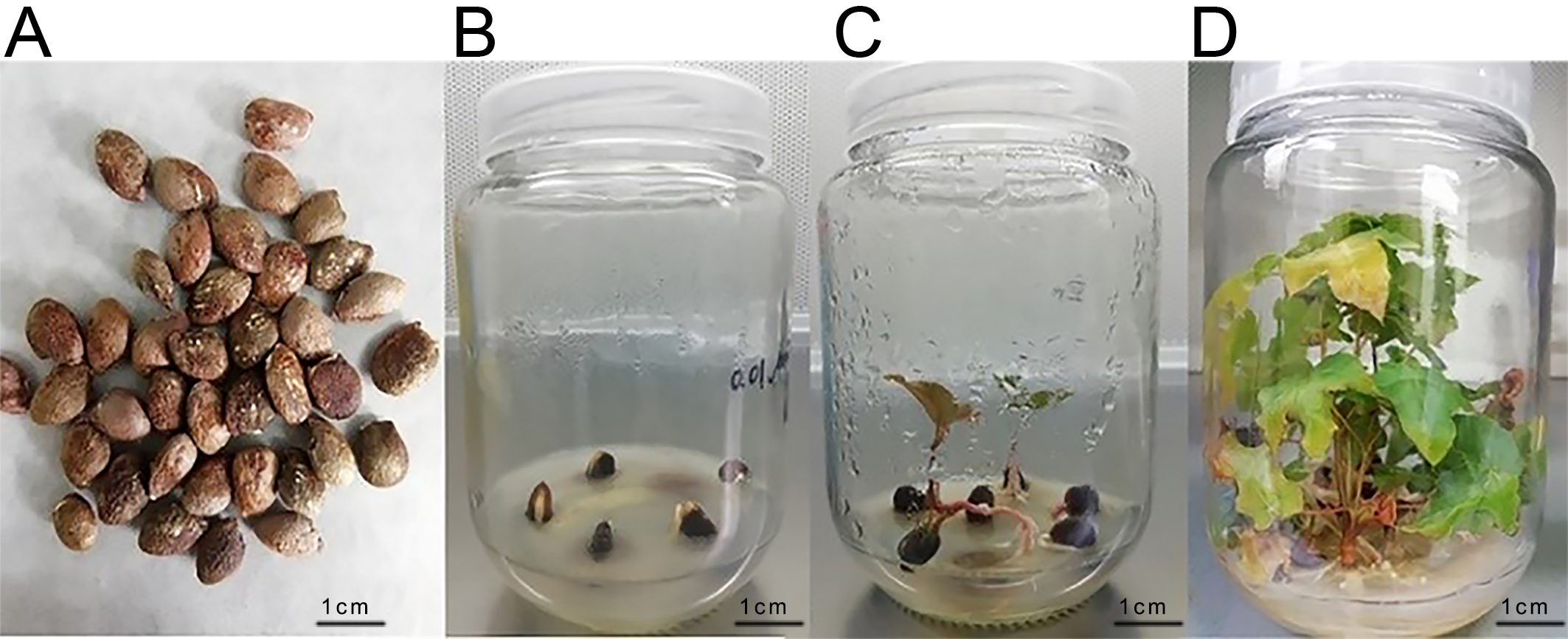

Supplement: Supplementary file 2 [file DataSheet2.zip › Supplementary Figure2.tif]

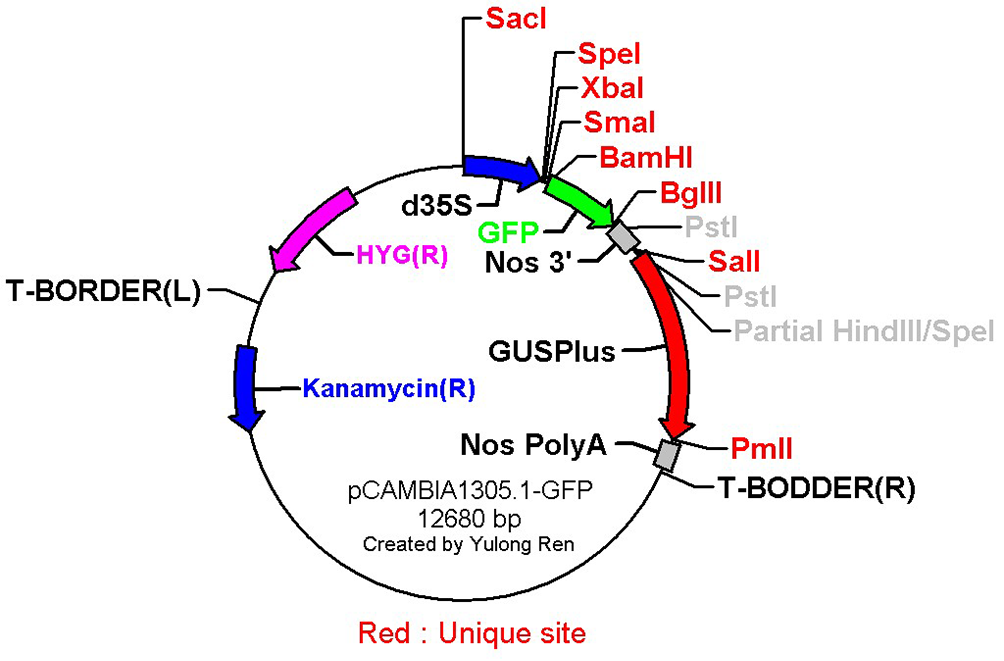

Supplement: Supplementary file 2 [file DataSheet2.zip › Supplementary Figure3.tif]

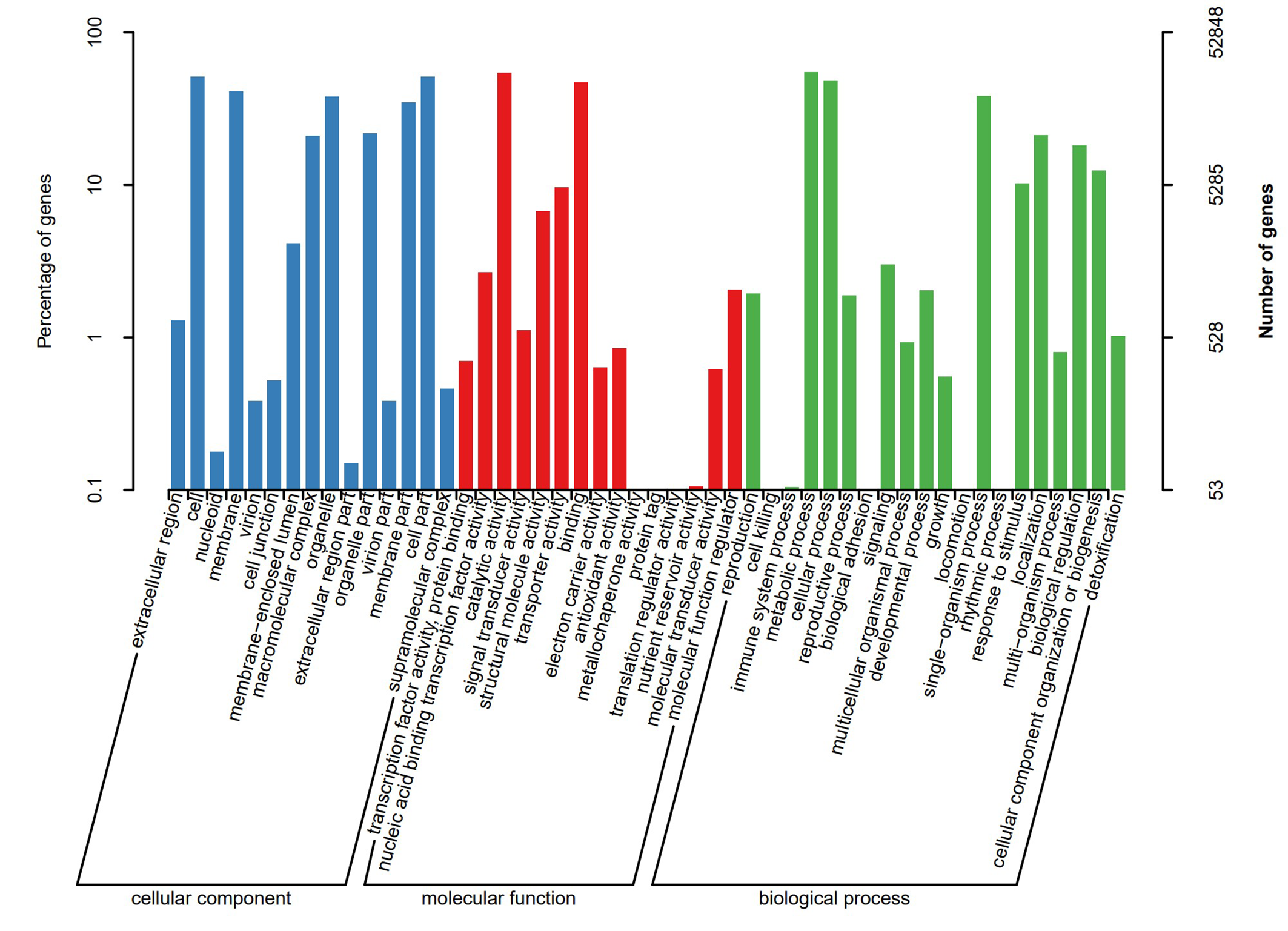

Supplement: Supplementary file 2 [file DataSheet2.zip › Supplementary Figure4.tif]

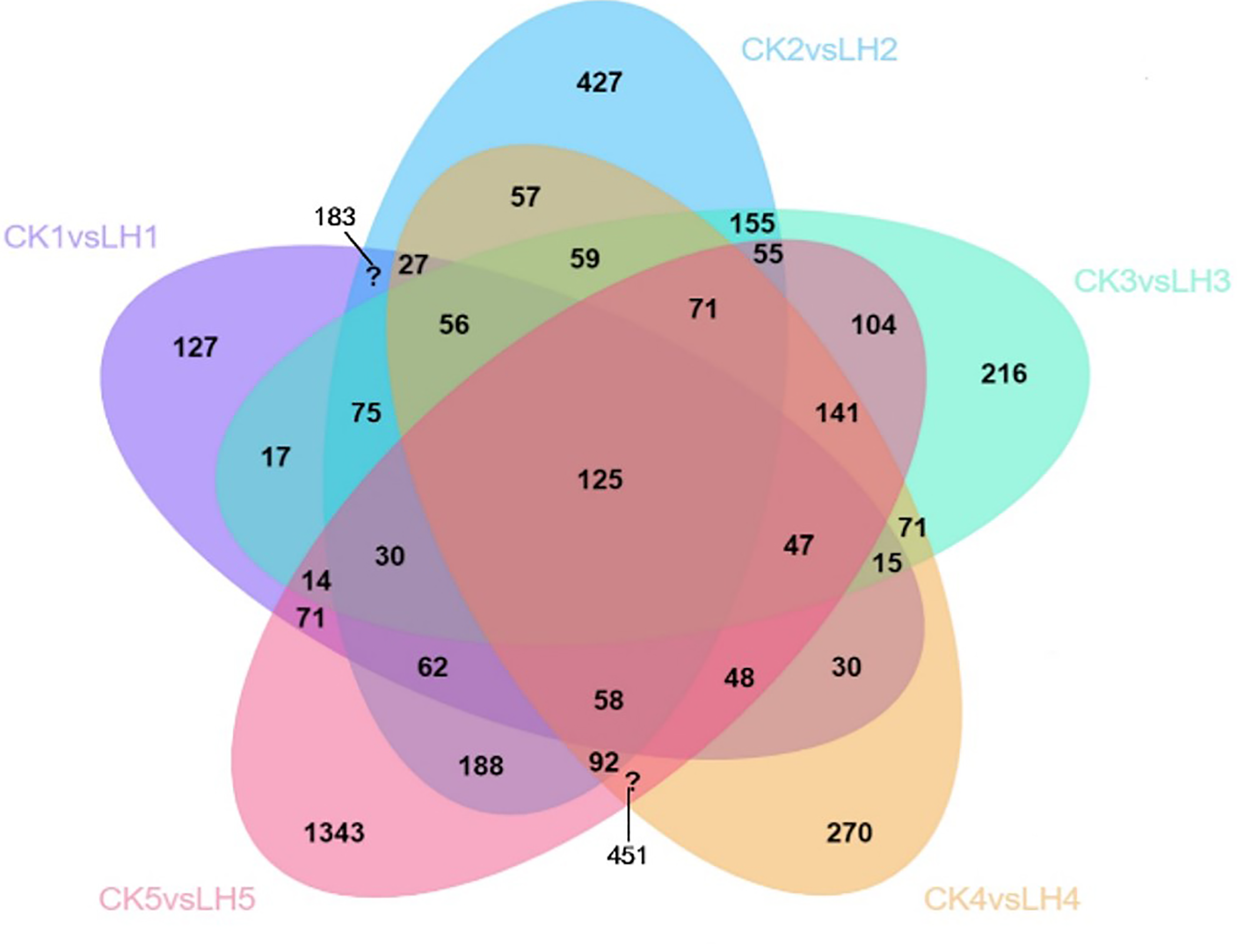

Supplement: Supplementary file 2 [file DataSheet2.zip › Supplementary Figure5.tif]

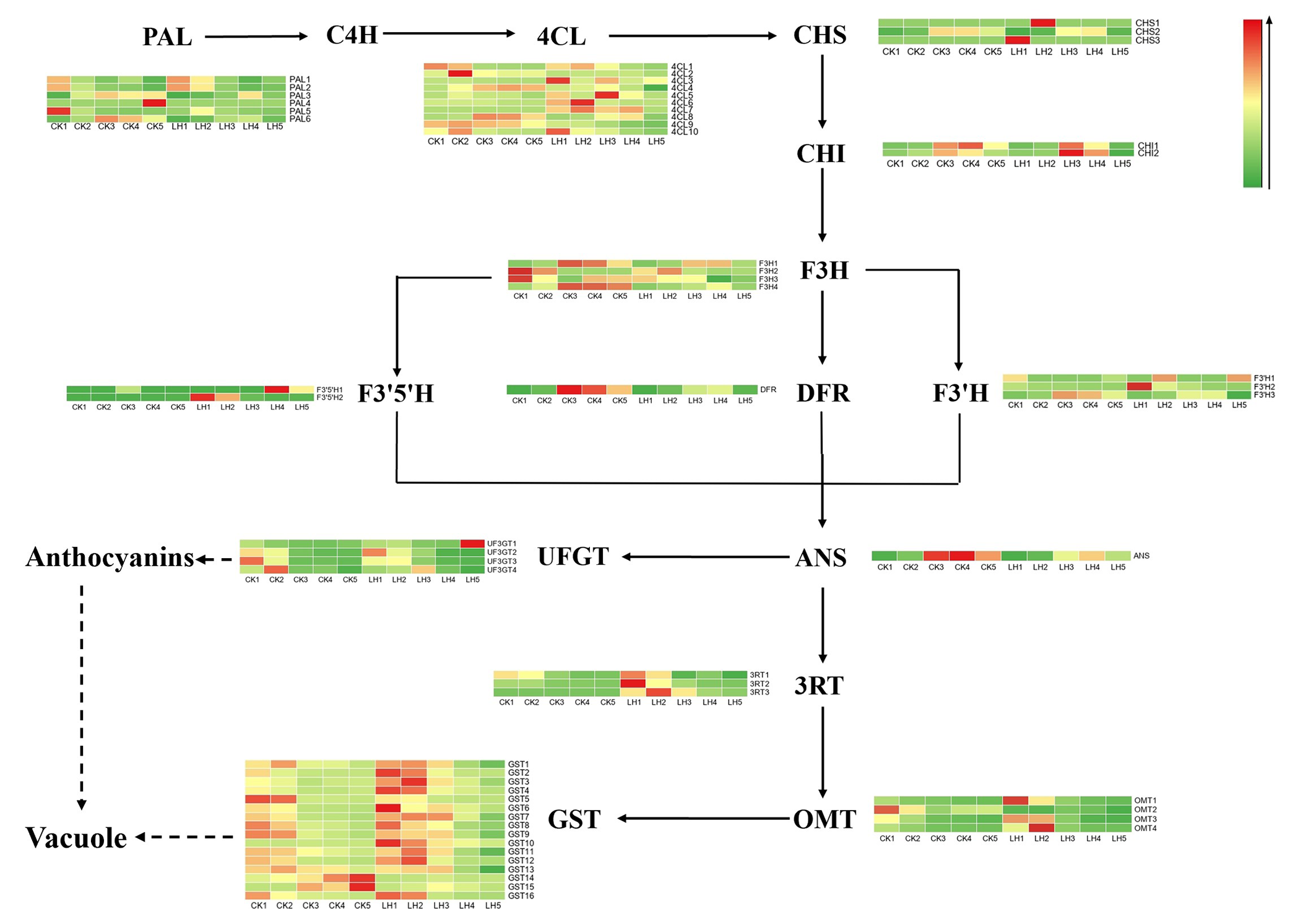

Supplement: Supplementary file 2 [file DataSheet2.zip › Supplementary Figure6.tif]
