# Supplementary material for: Preliminary establishment of genetic transformation system for embryogenic callus of Acer truncatum ‘Lihong’
Source: Front Plant Sci. 2024 Sep 5;15:1419313. doi: 10.3389/fpls.2024.1419313 (PMC11410635; doi:10.3389/fpls.2024.1419313)
Supplement: Supplementary file 3 [file Table1.docx]

Supplementary Material

Preliminary establishment of genetic transformation system for embryogenic callus of Acer truncatum Bunge

Yipeng Yang^1†^, Yuan Chan^2^ ^†^,Yongge Wang^1 †^, Hao guo^2^, Lina Song^1^, Huali Zhang^1^, Liping Sun^1^, Richen Cong^1^ and Hua Zhang ^1*^

*** Correspondence:** Hua Zhang: [seastory@163.com](mailto:seastory@163.com)

# Supplementary Tables

**SUPPLEMENTARY TABLE 1.** Composition of ½ MS basal medium

| Component | Quantity |
| --- | --- |
| MS Basal medium | 2.203 g |
| Sucrose | 30 g |
| Acid hydrolyzed casein | 0.5 g |
| ddH_2_O | up to 1000 mL |
| pH | 5.8 |

**SUPPLEMENTARY TABLE 2.** Composition of WPM basal medium

| Component | Quantity |
| --- | --- |
| WPM | 2.58 g |
| Sucrose | 30 g |
| Acid hydrolyzed casein | 0.5 g |
| ddH_2_O | up to 1000 mL |
| pH | 5.8 |

**SUPPLEMENTARY TABLE 3.** Concentrations of TDZ, 6-BA, IBA/IAA, and GA_3_ in embryonic callus induction medium

| Serial No. | TDZ (mg L^-1^) | 6-BA (mg L^-1^) | IBA/IAA (mg L^-1^) | GA_3_ (mg L^-1^) |
| --- | --- | --- | --- | --- |
| 1 | 1.0 | 3.0 | 0.1, 0.2, 0.4, 0.5, 1.0 | 0.1 |
| 2 | 1.0, 2.0, 3.0, 4.0, 5.0 | 3.0 | 0.5 | 0.1 |
| 3 | 1.0 | 1.0, 2.0, 3.0, 4.0, 5.0 | 0.5 | 0.1 |

**SUPPLEMENTARY TABLE 4** Pre-medium formula

| Test batches | Culture medium formula |
| --- | --- |
| 1 | WPM + 0.1 mol L^-1^ mannitol + 8g L^-1^ agar + 30 g L^-1^ sucrose |
| 2 | WPM + 30 g L^-1^ sucrose + 8 g L^-1^ agar + 0.5 g L^-1^ acid hydrolyzed casein + 0.5 mg L^-1^ KT + 2 mg L^-1^ 6-BA + 1.0 mg L^-1^ TDZ + 0.5 mg L^-1^ IBA + 0.1 mg L^-1^ GA_3_ |
| 3 | WPM + 30 g L^-1^ sucrose + 8 g L^-1^ agar + 0.5 g L^-1^ acid hydrolyzed casein + 0.2 mg L^-1^ KT + 1.0 mg L^-1^ TDZ + 0.5 mg L^-1^ IBA + 0.1 mg L^-1^ GA_3_ |

**SUPPLEMENTARY TABLE 5** Co-culture medium formula

| Test batches | Culture medium formula |
| --- | --- |
| 1 | WPM + 30 g L^-1^ sucrose + 8 g L^-1^ agar + 3 mg L^-1^ 6-BA + 1.0 mg L^-1^ TDZ + 0.5 mg L^-1^ IBA + 0.1 mg L^-1^ GA_3_ + 100 μmol L^-1^ AS |
| 2 | WPM + 30 g L^-1^ sucrose + 8 g L^-1^ agar + 0.5 mg L^-1^ KT + 2 mg L^-1^ 6-BA + 1.0 mg L^-1^ TDZ + 0.5 mg L^-1^ IBA + 0.1 mg L^-1^ GA_3_ + 100 μmol L^-1^ AS |
| 3 | WPM + 30 g L^-1^ sucrose + 8 g L^-1^ agar + 0.2 mg L^-1^ KT + 1.0 mg L^-1^ TDZ + 0.5 mg L^-1^ IBA + 0.1 mg L^-1^GA_3_ + 100 μmol L^-1^ AS |

**SUPPLEMENTARY TABLE 6** Antibacterial medium formula

| Test batches | Culture medium formula |
| --- | --- |
| 1 | WPM + 30 g L^-1^ sucrose + 8 g L^-1^ agar + 0.5 g L^-1^ acid hydrolyzed casein + 3 mg L^-1^ 6-BA + 1.0 mg L^-1^ TDZ + 0.5 mg L^-1^ IBA + 0.1 mg L^-1^ GA_3_ + 200 mg L^-1^ carbenicillin + 200 mg L^-1^ cephalosporin |
| 2 | WPM + 30 g L^-1^ sucrose + 8 g L^-1^ agar + 0.5 g L^-1^ acid hydrolyzed casein + 0.5 mg L^-1^ KT + 2 mg L^-1^ 6-BA + 1.0 mg L^-1^ TDZ + 0.5 mg L^-1^ IBA + 0.1 mg L^-1^ GA_3_ + 200 mg L^-1^ carbenicillin + 200 mg L^-1^ cephalosporin |
| 3 | WPM + 30 g L^-1^ sucrose + 8 g L^-1^ agar + 0.5 g L^-1^ acid hydrolyzed casein + 0.2 mg L^-1^ KT + 1.0 mg L^-1^ TDZ + 0.5 mg L^-1^ IBA + 0.1 mg L^-1^ GA_3_ + 200 mg L^-1^ carbenicillin + 200 mg L^-1^ cephalosporin. |

**SUPPLEMENTARY TABLE 7** Resistance screening medium formula

| Test batches | Culture medium formula |
| --- | --- |
| 1 | No |
| 2 | No |
| 3 | WPM + 30 g L^-1^ sucrose + 8 g L^-1^ agar + 0.5 g L^-1^ acid hydrolyzed casein + 3 mg L^-1^ 6-BA + 1.0 mg L^-1^ TDZ + 0.5 mg L^-1^ IBA + 0.1 mg L^-1^ GA_3_+ 300 mg L^-1^ carbenicillin+200 mg L^-1^ cephalosporin+ hygromycin(10 mg L^-1^, 15 mg L^-1^, 20 mg L^-1^ or 25 mg L^-1^) |

**SUPPLEMENTARY TABLE 8. Primers required for RT-PCR**

| Primer Name | Upstream Primer (5'-3') | Downstream Primer (5'-3') |
| --- | --- | --- |
| Actin | GGCTCAGTCTAAGCGTGGTA | GAACTGCTTGGATGGCAACA |
| *GST894* | GGATCCATGGCAGGCATCAAAAT | CTCGAGCTTCTTGCTTTGCAAAG |

**SUPPLEMENTARY TABLE 9. Statistical table of sequencing data quality**

| SampleID | ReadSum | BaseSum | GC(%) | Q20(%) | Q30(%) |
| --- | --- | --- | --- | --- | --- |
| CK1 | 21060906 | 6309670315 | 44.96 | 97.33 | 92.62 |
| CK2 | 20503223 | 6138599009 | 44.73 | 97.27 | 92.50 |
| CK3 | 21238133 | 6352194604 | 44.64 | 97.54 | 93.05 |
| CK4 | 23835267 | 7133118075 | 44.57 | 97.56 | 93.06 |
| CK5 | 22041093 | 6594937797 | 44.87 | 97.34 | 92.63 |
| LH1 | 22462139 | 6726444484 | 45.15 | 97.26 | 92.61 |
| LH2 | 20137758 | 6028025692 | 44.67 | 97.33 | 92.66 |
| LH3 | 21406328 | 6400903492 | 44.43 | 97.51 | 93.10 |
| LH4 | 22251392 | 6664123569 | 44.22 | 97.52 | 93.07 |
| LH5 | 21339819 | 6393250239 | 44.31 | 97.28 | 92.60 |

**SUPPLEMENTARY TABLE 10. Summary of the assembly results from transcriptome**

| Length range | Transcript | Unigene |
| --- | --- | --- |
| 200-300 | 50527（16.64%） | 42848（38.65%） |
| 300-500 | 38407（12.65%） | 25798（23.27%） |
| 500-1000 | 49195（16.20%） | 19666（17.74%） |
| 1000-2000 | 71021（23.39%） | 12806（11.55%） |
| 2000+ | 94442（31.11%） | 9754(8.80%) |
| Total Number | 303592 | 110872 |
| Total Length | 494435965 | 83676381 |
| N50 Length | 2680 | 1389 |
| Mean Length | 1628.62 | 754.71 |

**SUPPLEMENTARY TABLE 11 Summary of functional annotation of Unigenes**

| Functional annotation library | Annotated Number | 300<=length<1000 | length>=1000 |
| --- | --- | --- | --- |
| COG_Annotation | 22964 | 8000 | 8950 |
| GO_Annotation | 52848 | 20289 | 15585 |
| KEGG_Annotation | 21281 | 7724 | 8101 |
| KOG_Annotation | 34709 | 12608 | 12834 |
| Pfam_Annotation | 42477 | 15657 | 16974 |
| Swissprot_Annotation | 29878 | 10112 | 13528 |
| eggNOG_Annotation | 66697 | 25720 | 20212 |
| nr_Annotation | 71374 | 27855 | 20661 |
| All_Annotated | 73673 | 28377 | 20735 |
