# Supplementary material for: Preliminary establishment of genetic transformation system for embryogenic callus of Acer truncatum ‘Lihong’
Source: Front Plant Sci. 2024 Sep 5;15:1419313. doi: 10.3389/fpls.2024.1419313 (PMC11410635; doi:10.3389/fpls.2024.1419313)
Supplement: Supplementary file 4 [file Table2.docx]

Supplementary Material

Preliminary establishment of genetic transformation system for embryogenic callus of Acer truncatum Bunge

Yipeng Yang^1†^, Yuan Chan^2^ ^†^,Yongge Wang^1 †^, Hao guo^2^, Lina Song^1^, Huali Zhang^1^, Liping Sun^1^, Richen Cong^1^ and Hua Zhang ^1*^

*** Correspondence:** Hua Zhang: [seastory@163.com](mailto:seastory@163.com)

# Supplementary Supporting article

**1 woody plant**

**1.1 Leaf induced callus :**

1. **Establishment of genetic transformation system and isolation and identification of mirnas related to fruit color regulation of Ailanthus altissima‘ Liaohong’**

摘要：通过不同激素组合对‘聊红’椿愈伤组织诱导、芽诱导、根诱导的影响，得到最佳愈伤组织诱导培养基为在 MS 培养基上添加 0.1 mg/L 6-BA、0.1 mg/L 2,4-D、0.1mg/L NAA，嫩叶愈伤诱导率为 85.16%，生长情况最好。

- **Translation of relevant parts：**
- Abstract：Through the selection of explants, the best explants were tender leaves ; through the effects of different hormone combinations on callus induction, bud induction and root induction of *Ailanthus altissima*‘ Liaohong’, the best callus induction medium was obtained by adding 0.1 mg/L 6-BA, 0.1 mg/L 2,4-D and 0.1 mg/L NAA to MS medium. The callus induction rate of tender leaves was 85.16%, and the growth was the best.

1. **In vitro plant regeneration via callus culture of mature Salix exigua**

- Callus induction,callus growth,and plantlet regeneration were examined using leaf explants of three Salix exigua clones.Calli were initiated on three basal media supplemented with 0.1 mg/L (0.44 μM)or 0.5 mg/L (2.2 μM)of benzylaminopurine and 0.1 mg/L (0.45 μM)or 0.5 mg/L(2.3 μM)of 2,4-dichlorophenoxyacetic acid in a factorial fashion.

1. **Studies on callus tissue induction and subculture fromdifferent explants of *Quercus dentata***

- 2.1.1 槲树叶片诱导愈伤组织
- 以叶片为外植体，在含不同激素种类及浓度的不同培养基上培养25天，结果表明，４号处理的培养基诱愈伤导率最高（表3）。方差分 析 表 明，NAA对愈伤组织诱导具有显著影响（表4）。激素种类、激素浓度和培养基类型对槲树叶片愈伤组织诱导的影响按照重要性依次为NAA浓度＞培养基种类＞6-BA 浓度（表5），即NAA对叶片诱导愈伤组织作用最大。极差分析表明，最佳培养基为MS，NAA最佳浓度为1.00mg/L，6-BA最佳浓度为0.50 mg/L（表6）。综合激素、培养基种类对诱导率的影响结果，槲树叶片愈伤组织诱导的最佳培养基为 MS＋NAA 1.0 mg/L＋6-BA 0.5 mg/L。
- **Translation of relevant parts：**
- Using leaves as explants, they were cultured on different media containing various types and concentrations of hormones for 25 days. The results showed that medium number 4 had the highest callus induction rate (Table 3). ANOVA indicated that NAA had a significant effect on callus induction (Table 4). The influence of hormone type, hormone concentration, and medium type on the induction of callus from oak leaves, in order of importance, was NAA concentration > medium type > 6-BA concentration (Table 5), indicating that NAA had the greatest effect on the induction of callus from leaves. Range analysis revealed that the optimal medium was MS, with the best concentration of NAA being 1.00 mg/L and the best concentration of 6-BA being 0.50 mg/L (Table 6). Based on the combined effects of hormones and medium types on the induction rate, the optimal medium for the induction of callus from oak leaves was determined to be MS + NAA 1.0 mg/L + 6-BA 0.50 mg/L.

1. **Study on Tissue Culture Technology of Halophyte *Halimodendron halodendron* (Pall.) Voss**

2.4.2 外植体的愈伤组织诱导

将培养三周的无菌苗幼根、下胚轴、子叶、茎段分别取出，子叶切成 0.5cm×0.5cm的方块，幼根、下胚轴和茎段切成 0.5cm小段，分别接种于以MS+3%蔗糖+0.6%琼脂为基本培养基，添加不同质量浓度的 6-BA（0.1，0.5，1.0，1.5，2.0mg·L^-1^）和 2,4-D（0.5，1.0，1.5，2.0，4.0mg·L^-1^），共 25 个组合，每组设置10个重复，每个组培瓶中接种 3 个外植体，重复3次，分为光照培养组和暗培养组，直至 25天后记录每组培养基愈伤组织诱导率和愈伤组织状态。

幼茎诱导愈伤最适培养基为 0.5mg·L^-1^ 6-BA+0.5mg·L^-1^ NAA，诱导率100%；

**Translation of relevant parts：**

2.4.2 Callus Induction from Explants

- Aseptic seedlings were cultured for three weeks, after which the young roots, hypocotyls, cotyledons, and stem segments were taken out. The cotyledons were cut into 0.5 cm x 0.5 cm squares, and the young roots, hypocotyls, and stem segments were cut into 0.5 cm segments. These were inoculated into a basic medium consisting of MS + 3% sucrose + 0.6% agar, with the addition of different concentrations of 6-BA (0.1, 0.5, 1.0, 1.5, 2.0 mg/L) and 2,4-D (0.5, 1.0, 1.5, 2.0, 4.0 mg/L), forming 25 combinations in total. Each group had 10 replicates, with 3 explants inoculated per culture bottle, and repeated 3 times. The cultures were divided into a light culture group and a dark culture group. After 25 days, the callus induction rate and callus condition for each medium were recorded.
- The optimal medium for inducing callus from young stems was 0.5 mg/L 6-BA + 0.5 mg/L NAA, with an induction rate of 100%.

**1.2 Stem segment induced callus**

**1.** **Tissue culture of *Catalpa bungei* C.A.Mey and its active flavonoidcomposition in callus**

以MS、N6、DKW为基本培养基，添加不同浓度的6-BA和NAA，6-BA设置浓度1.0 mg L^-^¹、3.0 mg L^-^¹、5.0 mg L^-1^,NAA设置浓度0.01 mg L^-^¹、0.05 mg L^-^¹、0.10mg L^-^¹,辐照光质设置为白光、红光、蓝光，采用L9(4^3^)正交设计试验(具体处理组合见表3.1/3.4/3.7),共9种组合，另培养基中附加蔗糖30 g-L-,琼脂6.8 g L^-1^,pH调至5.8-5.9。取楸树无菌苗的叶片、叶柄、茎段为外植体接种于诱导愈伤组织的培养基上，每组接种10-12个外植体，重复3次，放置在各LED光源下培养，光照强度(PPFD)为50±5 μmol·m^-^².s^-^¹,光照时间为16h/d,温度为25±5℃。接种28天后观察各处理的愈伤组织生长情况，记录诱导情况。

茎段诱导愈伤组织的适宜配方为DKW+1.0 mg-L^-^¹6-BA+0.05 mg L^-^¹NAA，辐照光质为白光。

**Translation of relevant parts：**

- Using MS, N6, and DKW as the basic media, different concentrations of 6-BA and NAA were added. The concentrations of 6-BA were set at 1.0 mg/L, 3.0 mg/L, and 5.0 mg/L, and the concentrations of NAA were set at 0.01 mg/L, 0.05 mg/L, and 0.10 mg/L. The light qualities were set as white light, red light, and blue light. The experiment adopted an L9 (4³) orthogonal design (specific treatment combinations are shown in Tables 3.1/3.4/3.7), forming a total of 9 combinations. Additionally, the media were supplemented with 30 g/L sucrose and 6.8 g/L agar, with the pH adjusted to 5.8-5.9. Leaves, petioles, and stem segments from sterile Catalpa bungei seedlings were used as explants and inoculated onto the callus induction media. Each group was inoculated with 10-12 explants, with three replicates per group. The cultures were placed under different LED light sources, with a light intensity (PPFD) of 50±5 μmol·m-²·s-¹, a photoperiod of 16 hours per day, and a temperature of 25±5°C. After 28 days of inoculation, the growth of callus tissue in each treatment was observed and the induction situation was recorded.
- The optimal formulation for inducing callus from stem segments was DKW + 1.0 mg/L 6-BA + 0.05 mg/L NAA, with white light as the irradiation light quality.

**2. CallusInduction and GeneticTransformation of *Picea mongolica***

诱导愈伤组织最佳培养基的筛选：以沙地云杉成熟胚作为外植体，将外植体接种在 MS、WPM、DCR、LM、和 SH 五种基本培养基中，分别添加细胞分裂素 6-BA（0 和 0.5 mg·L^-1^）；生长素 NAA 为（0、0.1、0.3 和 0.5 mg·L^-1^），研究其对愈伤组织诱导的影响。培养基经过高压蒸汽灭菌后采用 9 cm 直径一次性培养皿分装，试验共 5 个处理，每个处理接种 5 个培养皿，每培养皿接种 10 个外植体，共 5个重复。添加蔗糖 20~30 g·L^-1^，琼脂 6 g·L^-1^，pH 值为 5.77~5.80，25±1℃暗培养，培养 7 d 之后开始观察愈伤组织的形成，20 d 后统计诱导率。

以无菌苗茎段为外植体诱导愈伤组织的最佳培养基组合为：DCR+6-BA 2.0 mg·L ^-1^+NAA 0.5 mg·L -1，诱导率达 95.33%。

**Translation of relevant parts：**

- Selection of the Optimal Medium for Callus Induction: Using mature embryos of Picea glauca as explants, the explants were inoculated into five different basic media: MS, WPM, DCR, LM, and SH, each supplemented with the cytokinin 6-BA (0 and 0.5 mg/L) and the auxin NAA (0, 0.1, 0.3, and 0.5 mg/L) to study their effects on callus induction. After autoclaving, the media were distributed into 9 cm diameter disposable culture dishes. The experiment included 5 treatments, with each treatment inoculating 5 culture dishes and each dish containing 10 explants, with a total of 5 replicates. The media were supplemented with 20-30 g/L sucrose and 6 g/L agar, and the pH was adjusted to 5.77-5.80. The cultures were incubated in the dark at 25±1°C. Callus formation was observed after 7 days of culture, and the induction rate was recorded after 20 days.
- The optimal medium combination for callus induction using aseptic seedling stem segments as explants was found to be DCR + 6-BA 2.0 mg/L + NAA 0.5 mg/L, with an induction rate of 95.33%.

**3.** **Research on Release of Seed Dormancy and the Establishment of Rapid Propagation System of Jatropha sinensis**

3.2.4 茎段诱导愈伤组织

3.2.4.1 不同培养温度对愈伤组织诱导的影响

将无菌苗茎段接种至 MS+6-BA1.0 mg/L+NAA1.0 mg/L 的培养基中，调节 pH 为5.8~6.0；再设置温度分别为 10℃、15℃、20℃、25℃、30℃，每个处理接种 30 株，重复处理 3 次，培养 14 d 后对比统计不同温度培养下愈伤组织诱导情况。

3.2.4.2 不同光照条件对愈伤组织诱导的影响

将无菌苗茎段接种至 MS+6-BA1.0 mg/L+NAA1.0 mg/L 的培养基中，调节 pH 为5.8~6.0；再设置两种条件分别培养，其一置于光照强度为 1800 Lx 的 24h 光照(QL)培养箱中，其二用黑色塑料袋包裹后置于 24h 黑暗(QD)的培养箱中进行对比培养。每个处理接种 30 株，重复处理 3 次。每 24h 统计愈伤组织诱导情况，培养 14 d 后对比统计不同光照培养下愈伤组织诱导情况。

3.2.4.3 不同基本培养基对愈伤组织诱导的影响

根据前面培养结果的筛选条件，将无菌苗茎段分别接种到 MS、WPM、B5 三种基本培养基上，各培养基中均添加激素 6-BA1.0 mg/L+NAA1.0 mg/L 的组合，以及蔗糖 30 g/L、琼脂 6 g/L，调节 pH 为 5.8~6.0。每个处理接种 30 株，重复处理 3 次。14d 后统计愈伤组织诱导率及愈伤生长状况。

3.2.4.4 不同生长调节剂配比对愈伤组织诱导的影响

根据前面培养结果的筛选条件，在诱导效果最好的培养基上进行植物生长调节剂组合。将无菌苗切成带 1~2 个腋芽的茎段作为外植体，在茎段侧面划出 1~2 个伤口，接种时注意将茎段伤口接触到培养基。分别接入不同植物生长调节剂和不同浓度的6-BA 0.5 mg/L、1.0 mg/L、1.5 mg/L 与 NAA 0.3 mg/L、0.5、mg/L、1.0 mg/L、1.5 mg/L组合以及蔗糖 30 g/L、琼脂 6 g/L，调节 pH 为 5.8~6.0 的培养基中诱导愈伤。每种处理接种 30 株，重复处理 3 次。14d 后统计愈伤组织诱导率及愈伤生长状况。

山桐子无菌苗茎段为外植体诱导愈伤组织最佳的培养基组合为：MS+6-BA1.0mg/L+NAA1.5mg/L，诱导率为66.67%，25℃、全光照条件为最佳培养条件。

**Translation of relevant parts：**

- 3.2.4.1 Effect of Different Culture Temperatures on Callus Induction
- Aseptic seedling stem segments were inoculated onto MS medium supplemented with 6-BA 1.0 mg/L and NAA 1.0 mg/L, with the pH adjusted to 5.8-6.0. The cultures were incubated at different temperatures of 10°C, 15°C, 20°C, 25°C, and 30°C. Each treatment involved 30 explants, with three replicates per treatment. After 14 days of culture, the callus induction rates at different temperatures were compared and analyzed.
- 3.2.4.2 Effect of Different Light Conditions on Callus Induction
- Aseptic seedling stem segments were inoculated onto MS medium supplemented with 6-BA 1.0 mg/L and NAA 1.0 mg/L, with the pH adjusted to 5.8-6.0. Two light conditions were set for comparison: one group was cultured in an incubator with a light intensity of 1800 Lx under 24-hour light (QL), and the other group was wrapped in black plastic bags and cultured in complete darkness (QD). Each treatment involved 30 explants, with three replicates per treatment. Callus induction was recorded every 24 hours, and after 14 days, the callus induction rates under different light conditions were compared.
- 3.2.4.3 Effect of Different Basic Media on Callus Induction
- Based on previous results, aseptic seedling stem segments were inoculated onto three different basic media: MS, WPM, and B5. Each medium was supplemented with 6-BA 1.0 mg/L, NAA 1.0 mg/L, 30 g/L sucrose, and 6 g/L agar, with the pH adjusted to 5.8-6.0. Each treatment involved 30 explants, with three replicates per treatment. After 14 days, the callus induction rates and callus growth conditions were recorded and compared.
- 3.2.4.4 Effect of Different Growth Regulator Combinations on Callus Induction
- Based on previous results, the best medium for callus induction was used to test different combinations of plant growth regulators. Aseptic seedling stem segments with 1-2 axillary buds were used as explants, with 1-2 wounds made on the sides of the stem segments. During inoculation, care was taken to ensure the wounds contacted the medium. Different combinations of growth regulators with varying concentrations of 6-BA (0.5 mg/L, 1.0 mg/L, 1.5 mg/L) and NAA (0.3 mg/L, 0.5 mg/L, 1.0 mg/L, 1.5 mg/L), as well as 30 g/L sucrose and 6 g/L agar, were tested, with the pH adjusted to 5.8-6.0. Each treatment involved 30 explants, with three replicates per treatment. After 14 days, the callus induction rates and callus growth conditions were recorded and compared.
- The optimal medium combination for callus induction from stem segments of aseptic seedlings of Dendropanax dentiger was found to be MS + 6-BA 1.0 mg/L + NAA 1.5 mg/L, with an induction rate of 66.67%, under conditions of 25°C and full light.

**2** **Aceraceae Juss：**

**2.1 Leaf induced callus :**

**1.A Study on Call us Induction and Organogenesis in Acer mono Maxim**

3.1.3愈伤组织的诱导

本实验对五角枫不同外植体诱导愈伤组织的产生时，选用MS基本培养基添加6-苄基嘌呤(6-benzyladenine,6-BA)、萘乙酸(a-naphthalene acetic acid,NAA)、2,4-D(2,4-二氯苯氧乙酸)三中激素，采用L₉(34)正交试验设计以考察不同激素种类和配比组合以及不同植物外植体对五角枫愈伤组织诱导的影响。外植体材料的来源是无菌苗的幼根，子叶，幼叶。无菌苗是由无菌体系中的种子在基本培养基MS中萌发长成的，培养周期即从种子成长为完整植株的时间为20天左右。培养条件：温度25±2℃,光照强度2200 Lux。

正交试验中采用的三种外植体的处理方式：叶片，将无菌苗的幼嫩叶片垂直于主脉分割成1 cm²的长条形；幼根，将无菌苗的根部的粗壮部位剪为长约2cm左右的根段，将无菌苗的子叶切割为0.5 cm²的小块，均按照正交设计表中的设计接种于不同激素组合的MS固体培养基上诱导愈伤组织。黑暗处理3周，每个水平40个重复，3周后观察统计愈伤生长情况和愈伤组织诱导率。3周后观察统计愈伤生长情况和各组织脱分化率。

五角枫愈伤组织诱导中以幼叶为最佳诱导材料。通过对三种激素的研究发现，五角枫外植体愈伤组织高效诱导的最优组合是6-BA浓度以0.5 mg/L,2,4-D浓度以1.0 mg/L,NAA浓度以0.5 mg/L

**Translation of relevant parts：**

- 3.1.3 Callus Induction
- In this experiment, the induction of callus from various explants of Acer mono was examined using MS basal medium supplemented with three hormones: 6-benzyladenine (6-BA), α-naphthalene acetic acid (NAA), and 2,4-dichlorophenoxyacetic acid (2,4-D). An L₉(3⁴) orthogonal design was used to investigate the effects of different hormone types and combinations, as well as different plant explants, on the induction of callus in Acer mono. The explant materials were derived from aseptic seedlings, including young roots, cotyledons, and young leaves. The aseptic seedlings were grown from seeds in a sterile system on MS basal medium, with a cultivation period of approximately 20 days from seed germination to complete plantlet formation. Cultivation conditions were maintained at a temperature of 25±2°C with a light intensity of 2200 Lux.
- The three types of explants were treated as follows in the orthogonal experiment: young leaves were cut into 1 cm² strips perpendicular to the main vein; young roots were cut into approximately 2 cm segments from the thick part of the root; cotyledons were cut into 0.5 cm² pieces. These explants were inoculated onto MS solid medium with different hormone combinations according to the orthogonal design table for callus induction. The cultures were kept in darkness for three weeks, with 40 replicates for each condition. After three weeks, the growth of the callus and the induction rate were observed and recorded. The dedifferentiation rate of the tissues was also evaluated.
- Among the explants, young leaves were found to be the best material for callus induction in Acer mono. The optimal combination of plant growth regulators for efficient callus induction was determined to be 6-BA 0.5 mg/L, 2,4-D 1.0 mg/L, and NAA 0.5 mg/L.

**2.2 Stem segment induced callus**

**1. Research on the Tissue Culture of *Acer griseum***

对血皮槭的营养器官嫩茎、叶和叶柄进行了愈伤组织的诱导研究。结果表明：各外植体中诱导愈伤组织的难易程度为嫩茎>叶柄>叶；2,4-D比NAA更有利于愈伤组织的形成，诱导血皮械产生愈伤组织的最佳培养基配方：改良MS+6-BA 1.8 mg/L+2,4-D 0.8 mg/L+0.6g/L活性炭，诱导率为80%

**Translation of relevant parts：**

Callus Induction Study on Nutritive Organs of Tender Stems, Leaves, and Petioles of Bletilla striata.A study was conducted to induce callus from the tender stems, leaves, and petioles of Bletilla striata. The results indicated that the ease of callus induction varied among the explants, with tender stems being the easiest, followed by petioles, and then leaves. Among the growth regulators tested, 2,4-D was found to be more effective than NAA for callus formation. The optimal medium for inducing callus in Bletilla striata was found to be modified MS medium supplemented with 1.8 mg/L 6-BA, 0.8 mg/L 2,4-D, and 0.6 g/L activated charcoal, achieving an induction rate of 80%.

**2. Establishment and optimization of tissue culture system of the stemsof *Acer pseudosieboldianum***

1.2.2.2 愈伤组织诱导培养

（1）IBA、6-BA 和 TDZ 不同浓度的筛选在预试验的基础上采用 3 因素 3 水平 L_9_(3^3^)的正交试验设计筛选 IBA、6-BA和 TDZ 的浓度。IBA 浓度分别为 0.30、0.40 和 0.50 mg·L^−1^，6-BA 浓度分别为 0.60、0.70 和 0.80 mg·L^−1^，TDZ 浓度分别为 0.10、0.15 和 0.20 mg·L^−1^。将消毒处理好的茎段分别接种于含有不同植物生长调节剂的 MS 培养基中。试验共 9 个处理，每个处理接种30瓶，每瓶培养基内接2-3个消毒好的茎段，重复3次，每 7 d 观察愈伤组织生长状况，30 d 后统计诱导率。

茎段诱导愈伤组织无需进行暗培养处理，愈伤组织诱导最佳组合为 MS+0.40mg·L^−1^ IBA+0.70mg·L^−1^ 6-BA+0.20mg·L^−1^ TDZ，诱导率最高，达69.44%；

**Translation of relevant parts：**

1.2.2.2 Callus Induction Culture

(1) Screening Different Concentrations of IBA, 6-BA, and TDZ

Based on preliminary experiments, a 3-factor 3-level orthogonal design (L_9_(3³)) was used to screen the concentrations of IBA, 6-BA, and TDZ. The concentrations tested were:

IBA: 0.30, 0.40, and 0.50 mg·L⁻¹

6-BA: 0.60, 0.70, and 0.80 mg·L⁻¹

TDZ: 0.10, 0.15, and 0.20 mg·L⁻¹

Sterilized stem segments were inoculated onto MS medium containing different combinations of these plant growth regulators. The experiment included 9 different treatments, each with 30 culture bottles. Each bottle contained 2-3 sterilized stem segments. The experiment was repeated 3 times. The growth of callus was observed every 7 days, and the induction rate was recorded after 30 days.

The dark culture was not required when stem segments were induced. The optimum combination of callus induction from stem segment was MS+0.40 mg·L^−1^ IBA+0.70mg·L^−1^ 6-BA+0.20 mg·L^−1^ TDZ, with the highest induction rate as 69.44%;

**2.3 Leaves and stem segments can induce callus**

**1. Effect of Plant Hormones on Callus Induction and Regeneration of Stem Segment and Leaf from *Acer negundo***

**2.1 2，4-D 浓度对叶片、茎段诱导愈伤组织效果**

从图 1 可见，低浓度 2，4-D(0.01 mg·L^-1^) 叶外植体出愈率和生根率相当，高达 70% 左右; 茎段外植体出愈率达90% 以上，但出根率不足20% 。随着 2，4-D 浓度增加两者外植体出愈率高达 100%，生根率逐渐降低至 0，愈伤组织质地也发生变化: 0.01mg·L^-1^出现绿色瘤状愈伤组织;0.05 mg·L^-1^的为乳黄颗粒状愈伤组织; 0.1mg·L^-1^的为水渍状或玻璃化愈伤组织。综上看出，愈伤组织的诱导 2，4-D浓度为0.01 mg·L^-1^较为适宜

**Translation of relevant parts：**

- 2.1 Effect of 2,4-D Concentration on Callus Induction from Leaf and Stem Explants
- As shown in Figure 1, at a low concentration of 2,4-D (0.01 mg·L⁻¹), the callus induction rate and rooting rate for leaf explants were approximately 70%, while the callus induction rate for stem explants was over 90%, but the rooting rate was less than 20%. As the concentration of 2,4-D increased, the callus induction rate for both types of explants reached 100%, but the rooting rate gradually decreased to 0. The texture of the callus also changed with different concentrations of 2,4-D: at 0.01 mg·L⁻¹, green tumor-like callus formed; at 0.05 mg·L⁻¹, yellow granular callus formed; and at 0.1 mg·L⁻¹, water-soaked or vitrified callus formed. Overall, a 2,4-D concentration of 0.01 mg·L⁻¹ was found to be most suitable for callus induction.

**2.Studies on induction and differentiation of callus andphysiological and biochemical basis ofroots of *Acer davidii* Franch**

生长调节剂对愈伤诱导的试验研究表明：TDZ 和 NAA 有利于叶片及茎段愈伤组织的诱导，最佳愈伤诱导培养基为：MS + TDZ 0.5mg/L + NAA 0.1 mg/L，愈伤组织出愈率分别达 98.7%和 93.2%，且叶片叶基部位较叶片中部和尖端出愈率高，褐化率低。

**Translation of relevant parts：**

TDZ and NAA benefit of the leaf and stem callus induction. The best callus inducation is MS + TDZ0.5mg/L + NAA 0.1 mg/L, and the callus were 98.7% and 93.2%. The leaf blade better the central part of leaf base, and less for browning rate.

***Acer Truncatum***

**Studies on callus culture and flavonoid formation of *Acer Truncatum* Bunge**

元宝枫叶片愈伤组织诱导的最佳条件：以 MS＋2,4-D 2.0 mg/L＋BA0.3mg/L＋NAA0.5mg/L＋PVP0.5%为培养基，在 3 月中旬～4 月中旬采集 2 年生或 10 年生的母树叶片为外植体，以叶片 0.5 ㎝ 2 大小，叶片正面朝上的接种方式接种，调节培养基pH 值为 5.8，愈伤组织诱导率可达 100%，生长旺盛。

**Translation of relevant parts：**

The optimum conditions for the callus induction in leaves are as follows:

MS medium supplemented with 2,4-D 2.0 mg/L，BA0.3mg/L, NAA0.5mg/L and PVP0.5% are suitable for the callus induction, the callus inductive rate can achieved 100% . The immature leaves collected from 2 year old or 10 year old tree from middle of March to April was the favorable for the callus induction. The optimal way of inoculation for the explants was that 0.5cm2 leaves’epidermis towards above and 1.0cm stem segment explants placed horizontally in the medium. The best pH value for the callus induction was 5.8.
